# Basic Self-disorders Across Psychiatric Diagnoses and Risk Syndromes: An Updated Meta-analysis

**DOI:** 10.1093/schbul/sbag047

**Published:** 2026-05-11

**Authors:** Andrés Estradé, Paolo La-Torraca-Vittori, Cecilia M Esposito, Farid Carranza, Joshep Revilla, Ilaria Basadonne, Valentina Floris, Ilaria Bonoldi, Stefano Damiani, Dominic Oliver, Giovanni Stanghellini, Josef Parnas, Paolo Fusar-Poli

**Affiliations:** Early Psychosis: Interventions and Clinical-detection (EPIC) Lab, Department of Psychosis Studies, Institute of Psychiatry, Psychology & Neuroscience, King’s College London, London SE5 8AF, United Kingdom; Department of Brain and Behavioral Sciences, University of Pavia, Pavia 27100, Italy; Department of Neurosciences and Mental Health, IRCCS Fondazione Ca’ Granda Ospedale Maggiore Policlinico, Milan 20122, Italy; Department of Psychiatry, Hospital Universitario, Universidad Autonoma de Nuevo Leon (UANL), Monterrey 64460, Mexico; Kent and Medway Medical School, University of Kent, Canterbury CT2 7FS, United Kingdom; Faculty of Medicine, Universidad Peruana Cayetano Heredia, Lima 15102, Peru; Department of Brain and Behavioral Sciences, University of Pavia, Pavia 27100, Italy; Department of Brain and Behavioral Sciences, University of Pavia, Pavia 27100, Italy; Department of Psychosis Studies, Institute of Psychiatry, Psychology & Neuroscience, King’s College London, London SE5 8AF, United Kingdom; South London and Maudsley (SLaM) NHS Foundation Trust, London SE5 8AZ, United Kingdom; Department of Brain and Behavioral Sciences, University of Pavia, Pavia 27100, Italy; Early Psychosis: Interventions and Clinical-detection (EPIC) Lab, Department of Psychosis Studies, Institute of Psychiatry, Psychology & Neuroscience, King’s College London, London SE5 8AF, United Kingdom; Department of Psychiatry, University of Oxford, Oxford OX3 7JX, United Kingdom; National Institute for Health Research (NIHR), Oxford Health Biomedical Research Centre, Oxford OX3 7JX, United Kingdom; Department of Health Sciences, University of Florence, Florence 50134, Italy; Centro de Estudios de Fenomenologia y Psiquiatrías, Diego Portales University, Santiago 8370109, Chile; Mental Health Centre Glostrup, Copenhagen University Hospital, Copenhagen 2600, Denmark; Center for Subjectivity Research, Department of Communication, University of Copenhagen, Copenhagen 2300, Denmark; Early Psychosis: Interventions and Clinical-detection (EPIC) Lab, Department of Psychosis Studies, Institute of Psychiatry, Psychology & Neuroscience, King’s College London, London SE5 8AF, United Kingdom; Department of Brain and Behavioral Sciences, University of Pavia, Pavia 27100, Italy; OASIS Service, South London and Maudsley (SLaM) NHS Foundation Trust, London SE5 8AZ, United Kingdom; National Institute for Health Research (NIHR), Maudsley Biomedical Research Centre, South London and Maudsley, London SE5 8AF, United Kingdom

**Keywords:** EASE, self-disorders, self-disturbance, minimal self, ipseity

## Abstract

**Background:**

Research on basic self-disorders (BSD) has expanded since the introduction of the Examination of Anomalous Self-Experience (EASE). Although originally formulated as characterizing schizophrenia spectrum disorders, EASE-defined BSD have been reported in other psychiatric conditions and in individuals at clinical high-risk for psychosis (CHR-P), raising questions about their diagnostic specificity and their distribution across diagnostic categories.

**Study Design:**

We conducted a Preferred Reporting Items for Systematic Reviews and Meta-Analyses- and Meta-analysis Of Observational Studies in Epidemiology-compliant meta-analysis of EASE studies in individuals with Diagnostic and Statistical Manual of Mental Disorders or International Classification of Diseases (DSM/ICD) mental disorders, CHR-P samples, and healthy controls. Random-effect meta-analyses examined weighted mean total and domain-specific EASE scores using binary and continuous scoring methods. Meta-regressions and sensitivity analyses assessed the distribution of BSD across 5 major diagnostic categories.

**Study Results:**

Thirty-eight studies were included in the quantitative synthesis (*n* = 858/318 patients and *n* = 130/97 healthy controls for binary/continuous EASE scores, respectively). Meta-regressions indicated that diagnostic categories accounted for a substantial proportion of between-study variance in total EASE scores (QM(4) = 98.64, *P* < .001, *R*^2^ = 0.703). Schizophrenia spectrum disorders (*k* = 21) showed markedly higher scores compared to healthy controls (*k* = 4, *P* < .001), other mental disorders (*k* = 8, *P* < .001), non-schizophrenia spectrum psychosis (*k* = 4, *P* < .001), and CHR-P individuals (*k* = 10, *P* = .007). CHR-P samples exhibited intermediate BSD levels, higher than other mental disorders (*P* = .002) and non-schizophrenia spectrum psychosis (*P* = .041).

**Conclusions:**

EASE-defined BSD show a more pronounced concentration within schizophrenia spectrum conditions and are also elevated in CHR-P individuals. Nevertheless, some forms of BSD might be present in other psychiatric conditions. Further longitudinal and methodologically harmonized research is needed to better clarify the developmental trajectories and clinical significance of BSD across diagnostic categories.

## Introduction

Since the early 2000s, there has been a reinvigorated interest and reformulation of classic phenomenological contributions to psychopathology, particularly for psychotic disorders. To a significant degree, this movement has been spearheaded by Parnas and Sass’ ipseity disturbance model (IDM),^1,2^ according to which schizophrenia spectrum disorders constitute clinically distinct entities within current psychiatric taxonomies characterized by disturbances at the most basic sense of the self (ie, basic self-disorders, or BSD). The basic self represents the ground foundation of all psychic experiences and selfhood^2^ and consolidates from early childhood.^3^ It is natural, automatic, pre-reflective (in contrast to the “reflective” narrative or autobiographical sense of self^4^) and constitutes the in-the-moment awareness that all experience articulates in first person perspective as “my” experience.^1,5^ This dimension describes a person’s spontaneous subjective experience to exist as a living being naturally and instinctively immersed in the surrounding world.^6^ The basic self is difficult to define as it emerges from a profound level, which implicitly confers a sense of self-presence and self-familiarity to all subjective experiences.^1^ According to the IDM, BSD represent both a clinical core and a risk phenotype of schizophrenia and related disorders, being present since early years of life and remaining stable over time, even after the resolution of acute psychotic symptoms.^7^

At an empirical research level, disturbances of the basic self can be measured with the Examination of Anomalous Self-Experience (EASE),^8^ a semi-structured interview requiring specialist training,^8^ which has demonstrated high internal consistency^9^ and good-to-excellent interrater reliability.^10-12^ Since its introduction in 2005, the EASE has become the gold standard for assessing BSD.^13^ While several self-rating questionnaires for the assessment of BSD have since been developed, none have properly been validated in relation to the EASE.^14^ Within the IDM, anomalous self-experiences assessed by the EASE are conceptualized as interrelated manifestations of a pervasive disturbance of subjectivity rather than as isolated symptoms. In this regard, a study on the diagnostic performance of the EASE in a mixed clinical sample of 226 participants reported that a cut-off EASE score of ≥11 was associated with high specificity for schizophrenia spectrum diagnosis.^15^

A growing literature has explored EASE-defined BSD phenomena in clinical samples other than schizophrenia-spectrum disorders. Emerging evidence suggests that at least some forms of EASE-defined BSD phenomena can be observed in non-schizophrenia spectrum psychosis,^1,16-19^ affective disorders,^20,21^ anxiety disorders,^22,23^ Aspergers’ syndrome,^24^ personality disorders,^25^ and clinical high-risk for psychosis samples (CHR-P).^11^ This has motivated ongoing discussion regarding the diagnostic specificity—or degree of specificity—of BSD for schizophrenia, their potential transdiagnostic distribution, and the broader relevance of disturbances of self-experience across multiple psychopathological domains.^26,27^

Previous reviews and meta-analysis have assessed BSD across mental disorders, reporting a marked hyperaggregation in schizophrenia spectrum conditions, when assessing total or overall scores of BSD.^13,17,28-30^ While some previous studies focused on EASE-defined BSD,^13,17,28^ other combined findings from the EASE with conceptually related but non-equivalent instruments.^29,30^ Since then, several new empirical studies employing the EASE have been published in both mental disorders and psychosis risk syndromes.^31-41^ Against this background, the present review and meta-analysis was designed to provide an updated meta-analytic synthesis of the distribution of EASE-defined BSD across specific psychiatric diagnoses, as well as major diagnostic categories. This study focused on the EASE as the gold-standard assessment tool, and considered both total and domain-specific binary and continuous EASE scores. In addition, we included risk syndromes for severe mental disorders, which had only been included in a 2021 meta-analysis.^29^ Moreover, we draw on selected principles from the TRANSD framework^42,43^ to guide transparent reporting and structured appraisal of primary studies. In particular, we emphasize (1) explicit reporting of diagnostic definitions and assessment procedures, (2) comparative evaluation of BSD across clearly defined diagnostic groups, (3) examination of robustness across scoring approaches, and (4) consideration of the current degree of external validation.

According to the IDM, the EASE was developed to capture a unified disturbance of subjectivity. In this sense, a recent study reported a predominantly monofactorial structure based on a reduced 20-item version of the instrument, largely derived from a schizophrenia spectrum sample.^15^ As such, the extent to which these results generalize to the full 57-item EASE or to diagnostically heterogeneous samples remains an open question. In this study, domain-level distribution of BSD was included for descriptive and exploratory purposes, to examine potential differential patterns of anomalous self-experience across diagnostic groups, without assuming theoretically independent domains.

Overall, the present study aims to provide an updated and methodologically refined synthesis of EASE evidence and to evaluate claims of transdiagnostic distribution of BSD. In the context of the present study, we operationalize “transdiagnostic distribution” in a descriptive sense, referring to the presence and comparative magnitude of EASE-defined BSD across different diagnostic categories. This operationalization does not presuppose a shared underlying mechanism or an equal distribution across diagnoses, but rather examines the extent to which BSD are empirically detectable beyond the schizophrenia spectrum. This could have implications for the ongoing debate regarding categorical vs dimensional approaches to disorder classification, as well as provide valuable input to improve the predictive accuracy of assessments of individuals at risk for severe mental disorders.^44^

## Methods

### Design and Objective

We conducted a meta-analysis of EASE-defined BSD across Diagnostic and Statistical Manual of Mental Disorders or International Classification of Diseases (DSM/ICD)-defined mental disorders, risk syndromes, and healthy controls, in accordance with the 2020 Preferred Reporting Items for Systematic Reviews and Meta-Analyses^45^ and the Meta-analysis Of Observational Studies in Epidemiology guidelines^46^ (Tables S1 and S2). This review was not prospectively registered in a protocol database; analytic decisions were finalized after screening and data extraction due to the heterogeneity of outcome reporting. The application of TRANSD principles^42,43^ within the present study is summarized in Supplementary material (Table S3). The objective of this review was to provide an updated descriptive synthesis of EASE-defined BSD scores across diagnostic groups and to quantitatively compare their distribution using meta-analytic methods.

### Search Strategy and Study Selection

We searched PubMed and Web of Science (including: WoS Core Collection, MEDLINE, SciELO Citation index, Preprint Citation Index, and ProQuest Dissertations & Theses Citation index) for all records from inception until 31^st^ July 2025 using the following terms in title/abstract: (self-disorder^*^ OR “anomalous self-experience” OR ipseity OR “basic self” OR self-disturbance) AND (psychiatr^*^ OR “mental disorder” OR psychopathology). First, three independent researchers (AE, PLTV, CME) screened all results based on title and abstract. Eligible records were then screened at the full-text level against the inclusion and exclusion criteria by four independent researchers (AE, PLTV, CME, FC). Each full-text record was assessed by at least two independent researchers, and disagreements were resolved via a consensus call.

Inclusion criteria were: (1) empirical (observational, experimental, or clinical) studies providing quantitative information on BSD, measured by the EASE^8,10^; (2) published in peer-reviewed journals; (3) including samples with a DSM/ICD-any version diagnosis of any axis-I or axis-II mental disorder, ascertained clinically or with validated psychometric instruments, or including samples with risk syndromes for severe mental disorders, encompassing the clinical high risk state for psychosis (ie, CHR-P: Comprehensive Assessment of At Risk Mental State^47^; Semistructured Interview for Psychosis-Risk Syndromes^48^), clinical high risk state for bipolar (ie, Semistructured Interview for Bipolar At Risk States^49^); (4) including an overall sample of at least *n* = 10 individuals; (5) published in any language. Although the presence of a healthy control group was not an inclusion criterion, healthy control groups (as operationalized by each primary study) were additionally retrieved when available. We excluded secondary or other non-primary reports, studies not reporting quantitative EASE scores, and studies on BSD not using the EASE. However, secondary studies retrieved as part of the literature search were manually screened to identify additional suitable studies or data inaccessible via primary papers.

### Strategy for Data Extraction and Synthesis

Data were extracted for each study by at least 2 independent researchers (AE, PLTV, CME, FC): author and publication year, country, clinical diagnosis, sample size, female gender (*n* and %), sample age mean (SD or range), EASE scoring method (see below), and mean and SD of the EASE scores, when available. To minimize data loss, we scrutinized associated publications to retrieve additional data if the primary study reported incomplete quantitative data (eg, missing SD). If required, we derived the SD of EASE scores from the standard error or range.^50^

The EASE allows a binary or continuous scoring method for both total EASE and domain-specific scores.^8^ In the binary scoring method, BSD are rated for their frequency as “present” or “not present” (the frequency indexes the count of BSD detected): one point in the EASE indicates the presence of one BSD so that the resulting score indexes the cumulative frequency of BSD (total EASE scores ranging from 0 to 57, or 0 to 88 if subitems are included). In the continuous scoring method, BSD are rated on a 5-point scale based on their combined frequency and severity: 0 = absent; 1 = questionably present; 2 = definitely present, mild; 4 = definitely present, moderate; 5 = definitely present, severe. The EASE employs the binary/continuous scoring to produce both total score or domain-specific scores (5 domains: *Cognition and stream of consciousness* [17 items], *Self-awareness and presence* [18 items], *Bodily experiences* [9 items], *Demarcation/transitivism* [5 items], and *Existential reorientation* [8 items]^16^). The EASE does not provide any pre-defined thresholds for low or high EASE scores.^8^ Our meta-analyses accommodate both binary and continuous and total and domain-specific scoring, excluding subitems. We planned meta-analyses investigating both the frequency alone (binary scoring method) and the cumulative frequency and severity (continuous scoring method) of baseline BSD across DSM/ICD-any version diagnosis of any axis-I or axis-II mental disorder, risk syndromes, and healthy control groups. The effect size was the weighted mean using a random-effects model (ie, with each study being assigned a weight based on the inverse of the within-study variance plus between-study variance)^51^ of the baseline EASE score, as reported by each primary study. Heterogeneity was addressed with the *I*^2^ statistic.^51^

We stratified psychotic samples into two major categories: “schizophrenia-spectrum (SCZS) psychotic disorders” (ie, schizophrenia, schizophreniform, schizoaffective disorder), and all other “non-SCZS psychotic disorders” (ie, any depressive or bipolar disorder with psychotic features, delusional disorder, brief psychotic disorder, acute polymorphic psychotic disorder [APPD], substance-induced psychotic disorder, and psychotic disorder not otherwise specified [NOS]). Studies were excluded from the meta-analysis if they reported overlapping samples, used a scoring method other than the standard binary or continuous methods previously described (eg, included subitems in the scoring), had less than 5 participants in the diagnostic group, or used a mixed clinical sample definition that did not allow for diagnosis-specific estimates (eg, mixing SCZS and non-SCZS psychotic disorders, or mixed clinical samples). All analyses were performed in R version 4.2.1^52^ with the “metafor” package.^53^

In sensitivity analyses, we first stratified participants across five major diagnostic categories: “SCZS disorders” (ie, schizophrenia, schizophreniform, schizoaffective disorder, and schizotypal disorder), “non-SCZS psychosis,” “other mental disorders” (ie, any other non-psychotic DSM/ICD-any version diagnosis), “risk syndromes,” and “healthy controls.” Meta-regression tested between-group differences of the total binary EASE scores across these major diagnostic categories (SCZS disorders, non-SCZS psychosis, other mental disorders, risk syndromes, and healthy controls). Between-group differences were assessed with the Q statistic, and the proportion of between-study heterogeneity explained was addressed with the *R*^2^ index.^51^ In addition, we tested if group differences in pairwise comparisons survived after selecting only higher-quality studies that: (1) employed a psychopathology-focused observational design (ie, excluding studies purposely recruiting participants for electroencephalogram [EEG], magnetic resonance imaging or other type of experimental protocols); (2) recruited participant from community-based healthcare services; (3) screened for schizotypal traits in non-psychotic and non-CHR-P samples (not applicable for healthy control samples); and (4) employed measures to enhance the reliability of EASE ratings (ie, adequate EASE training or supervision of raters, reporting of inter-rater reliability scores, or consensus meetings to discuss and review EASE ratings). Finally, an exploratory meta-regression examined whether study region (Denmark vs non-Denmark) accounted for variability in schizophrenia-spectrum EASE estimates.

Comparisons across diagnostic groups were performed in a descriptive and exploratory framework. Given the limited number of studies and sample sizes for several of the diagnostic groups, formal correction for multiple comparisons was not applied. Consequently, results are to be interpreted primarily based on effect sizes (weighted mean scores), confidence intervals, and consistency of patterns, rather than binary significance thresholds.

### Quality Analysis

The overall methodological quality of studies included in the meta-analysis of weighted mean EASE scores was assessed using an adapted version of the risk of bias tool for epidemiological studies by Hoy et al.^54^ (Table S4). Each item rated as “Yes” is granted one point. Items rated as “No” or “Can’t tell,” are granted 0 points (maximum possible score of 10).

## Results

### Database

**Figure 1:**
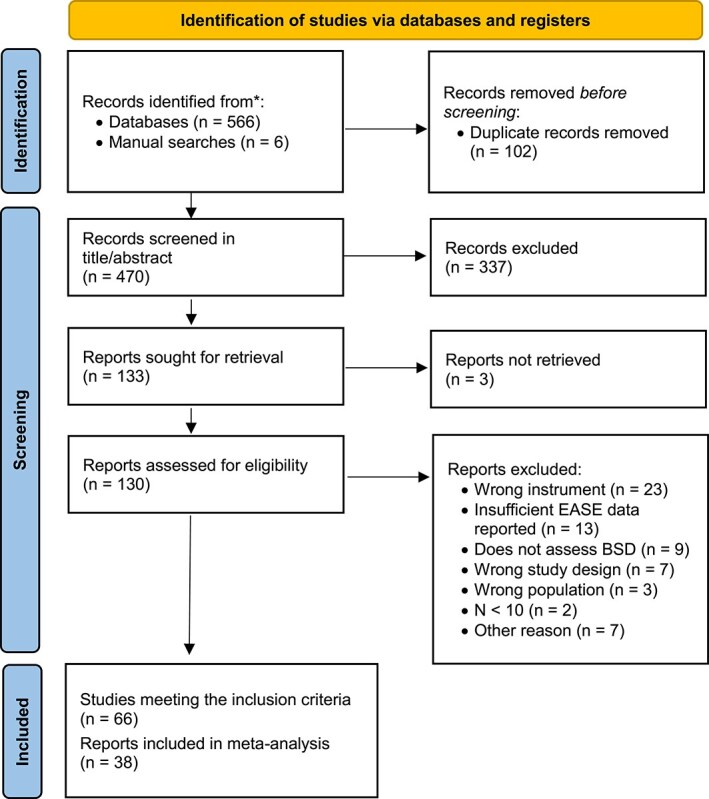
PRISMA 2020 Flow Chart for the Systematic Review and Meta-analysis.

The literature search retrieved 566 records after duplicates removal, plus 6 via manual searches (Figure 1). Following title/abstract and full-text screening, we retrieved 66 studies providing quantitative scores on EASE-defined BSD in clinical populations. Of these, 28 were excluded from the meta-analysis, mainly due to overlapping samples (Table S5). Therefore, we conducted our meta-analysis on the remaining 38 studies^9,11,15,22,24,25,33,37,39,41,55-82^ (Table 1). All studies were observational and predominantly focused on psychopathological explorations. A subset of studies incorporated laboratory tasks, including EEG,^41,56,65,71^ neuroimaging,^77^ gait and posture analysis,^33^ and linguistic analysis.^40^ Eight studies conducted neurocognitive assessments.^56,58,61,64,67,68,70,74^ No interventional clinical studies were found. The studies were conducted in Australia (*k* = 7), Austria (*k* = 1), Denmark (*k* = 10), France (*k* = 1), Israel (*k* = 2), Italy (*k* = 7), Norway (*k* = 5), Portugal (*k* = 2), Slovenia (*k* = 1), Switzerland (*k* = 1), and Republic of Korea (*k* = 1). We retrieved EASE scores for 17 distinct samples (*n* ≥ 5) of SCZS psychotic disorders,^25,33,37,40,41,55,57,60,61,64,65,71,74,76,82^ 16 for CHR-P individuals,^11,36,38,56,59,66-70,73,77,81,83,84^ 6 for schizotypal disorder,^9,24,25,60,63,73^ 5 for non-SCZS spectrum psychosis (ie, APPD, bipolar or affective disorder with psychotic features, delusional disorder, substance-induced psychotic disorder, psychosis NOS),^41,55,58,82^ and single samples for borderline personality disorder,^38^ Asperger’s syndrome,^24^ affective disorders (ie, major depressive disorder or cyclothymia),^60^ obsessive-compulsive disorder (OCD),^62^ and panic disorder.^22^ In addition, five samples were only included in the major diagnostic categories analyses: 1 for schizophrenia spectrum disorders^39^ and 4 for other mental disorders^9,63,69,70^. We also retrieved EASE scores for 7 distinct samples of healthy controls.^11,22,58,64,75,77,84^ Most studies (*k* = 28, 71.79%) used the binary EASE scoring method, 8 (20.51%) used the continuous method, and 3 (7.69%) provided scores using both methods. Quality assessment of the 38 studies included in the meta-analysis ranged from 4 to 9 (Median = 7, Table S6). Thirty samples with binary EASE scores, from 18 studies,^9,22,24,25,36,39,60,61,63,64,67,69,70,73,75,76,81,82^ met our high methodological-quality criteria to be included in sensitivity analyses.

**Table 1:** Primary Studies Investigating Basic Self-Disorders Using the EASE Included in the Meta-analysis

Study author(s) and publication year	Country	Samples (*n*) [reason for exclusion from meta-analyses]	Female gender, *n* (%)	Mean age (SD) [range]	Group definition	Design (risk of bias score – max. 10)	Study’s main focus
Binary EASE scoring method							
Gruber 2023^38^	Austria	**- CHR-P: UHR (*n* = 24)** **- Borderline personality disorder (*n* = 27)** - Mixed psychotic disorders: FEP (*n* = 29) [group definition]	12 (50) 25 (92.6) 15 (51.7)	22.55 (2.97) 28.40 (6.49) 24.15 (3.70)	DSM-IV, CAARMS	Observational, cross-sectional (7)	Psychopathology (personality functioning)
Davidsen 2009^59^	Denmark	**- CHR-P: UHR (*n* = 11)**	6 (54.54)	23.3 [18-28]	SIPS	Observational, cross-sectional (5)	Psychopathology
Handest 2025^39^	Denmark	**- Schizophrenia spectrum disorders (*n* = 62)** ^**a**^	.	.	ICD-10	Observational, cross-sectional (7)	Psychopathology and social functioning
Raballo and Parnas 2012^60^	Denmark	**- Schizophrenia spectrum psychosis (*n* = 19)** **- Schizotypal disorder (*n* = 8)** **- Affective disorders (*n* = 9)**	12 (63.16) 2 (25) 5 (55.56)	25.7 (6.4) 28.0 (4.6) 27.3 (8.3)	ICD-10	Observational, cross-sectional (7)	Psychopathology
Nordgaard and Parnas 2014^9^	Denmark	**- Schizotypal disorder (*n* = 22)** **- Bipolar disorder, major depression, anxiety disorders, OCD, and non-SPD personality disorders (n = 32)** ^**a**^ - Non-affective psychosis (*n* = 46) [group definition]	18 (81.82) 19 (59.38) 29 (63.04)	25 [19-47] 31.22 [18-60] 26.5 [18-59]	DSM-IV	Observational, longitudinal (8)	Psychopathology
Nordgaard 2015^61^	Denmark	**- Schizophrenia spectrum psychosis (*n* = 31)**	16 (51.61)	25.2 [18-37]	DSM-IV	Observational, cross-sectional (8)	Psychopathology and neurocognition
Nilsson 2020^24^	Denmark	**- Asperger’s syndrome (*n* = 22)** **- Schizotypal disorder (*n* = 29)**	5 (23) 14 (48)	23.1 (3.99) 23.2 (2.46)	ICD-10	Observational, cross-sectional (8)	Psychopathology
Rasmussen 2020^62^	Denmark	**- OCD (*n* = 12)** ^b^ - Schizotypal disorder (*n* = 14) [overlapping] - Non-affective psychosis (*n* = 12) [group definition]	– – –	– – –	DSM-5	Observational, cross-sectional (8)	Psychopathology
Zandersen and Parnas 2020^25^	Denmark	**- Schizophrenia spectrum psychosis (*n* = 6)** **- Schizotypal disorder (*n* = 14)** - Borderline personality disorder (*n* = 3) [*n* < 5] **-** Other diagnoses (*n* = 7) [group definition]	– – – –	– – – –	DMS 5	Observational, cross-sectional (7)	Psychopathology
Rasmussen 2022^63^	Denmark	**- Schizotypal disorder (*n* = 15)** **- OCD (*n* = 12) + MD (*n* = 4)** ^**a**^ - Non-affective psychosis (*n* = 32) [group definition]	10 (66.67) 13 (81.25) 21 (65.63)	27.3 (5.8) 32 (7.2) 30.1 (6.8)	DSM-5	Observational, cross-sectional (8)	Psychopathology
Sandsten 2022^64^	Denmark	**- Schizophrenia spectrum psychosis (*n* = 35)** **- Healthy controls (*n* = 35)**	21 (60) 21 (60)	22.11 (3.932) 24.06 (3.24)	DSM-5	Observational, cross-sectional (8)	Psychopathology and neurocognition
Martin 2017^65^	France	**- Schizophrenia spectrum psychosis (*n* = 28)**	5 (17.86)	31.0 (7.9)	DSM-5	Observational, cross-sectional (5)	EEG task (temporal prediction)
Koren 2016^66^	Israel	**- CHR-P: APS, non-help-seeking (*n* = 12)** - Non-APS control sample (*n* = 88) [group definition]	8 (63) 53 (60)	13.9 (0.7) 14.0 (0.9)	DSM 5, SIPS	Observational, cross-sectional (6)	Psychopathology
Koren 2019^67^	Israel	**- CHR-P: APS (*n* = 21)** - Mixed clinical sample (*n* = 40) [group definition]	9 (42.9) 18 (43.9)	15.8 (1.4) 15.9 (1.5)	SIPS	Observational, cross-sectional (8)	Psychopathology, neurocognition, and metacognition
Monducci 2025^81^	Italy	**- CHR-P: UHR (*n* = 33)** - Mixed psychotic disorders: FEP (*n* = 17) [group definition] - Clinical help-seeking controls (*n* = 45) [group definition]	22 (65.7) 11 (62.5) 25 (56.8)	15.18 (1.48) 16.12 (1.40) 15.40 (1.35)	SIPS	Observational, cross-sectional (7)	Psychopathology (suicidality)
Raballo 2016^69^	Italy	- **CHR-P: UHR and COPER/COGDIS (*n* = 29)** **- Non-CHR (personality [*n* = 3] + anxiety [*n* = 5] + mood [*n* = 2] + other disorders [*n* = 8])**^**a**^	8 (25.81) 9 (50)	20.32 (2.84) 20.05 (3.29)	DSM-IV, SIPS, SPI-A/CY	Observational, cross-sectional (8)	Psychopathology
Raballo 2018^70^	Italy	**- CHR-P: UHR (*n* = 23)** **- Mixed non-psychotic clinical sample (*n* = 60)** ^**a**^ **-** Non-affective psychosis (*n* = 13) [group definition]	12 (52.2) 38 (63.3) 8 (61.5)	15.6 (1.2) 15.5 (1.3) 15.7 (1.6)	DSM-IV, SIPS	Observational, cross-sectional (8)	Psychopathology and neurocognition
Lucarini 2024^40^	Italy	**- Schizophrenia spectrum psychosis (*n* = 29)**	11 (37.9)	35.52 (12.73)	DSM-5	Observational, cross-sectional – linguistic analysis (6)	Linguistic analysis (turn-taking)
Tonna 2023^33^	Italy	**- Schizophrenia spectrum psychosis (*n* = 43)**	11 (25.58)	35.5 (12.8)	DSM-5	Observational, cross-sectional—(6)	Gait and posture analysis
Haug 2012^82^	Norway	- **Bipolar psychosis (*n* = 21)** **- Other psychosis (13)** - **Schizophrenia spectrum psychosis (*n* = 57)**	13 (62) 2 (15) 28 (49)	24.4 (7.7) 23.8 (6.9) 25.4 (7.3)	DSM-IV	Observational, cross-sectional (9)	Psychopathology
Fischer-Vieler 2025^37^	Norway	**- Schizophrenia spectrum psychosis, with history of severe violence (*n* = 17)** **- Schizophrenia spectrum psychosis, without history of severe violence (*n* = 16)**	0 (0%) 0 (0%)	35.1 (10.4) 34.6 (6.9)	DSM-IV	Observational, cross-sectional (6)	Psychopathology (violent behavior)
Haug 2023^74^	Norway	**- Schizophrenia spectrum psychosis (*n* = 35)** ^c^	18 (51.4)	25.5 (7.8)	DSM-IV	Observational, longitudinal (9)	Psychopathology and neurocognition
Madeira 2017^22^	Portugal	**- Panic disorder (*n* = 47)** ^d^ **- Healthy controls (*n* = 47)** ^d^	32 (68.1) 28 (59.6)	38.17 (13.66) 39.09 (16.61)	ICD-10	Observational, cross-sectional (6)	Psychopathology
Madeira 2019^75^	Portugal	**- Healthy controls (*n* = 24)** - Mixed psychotic disorders: FEP (*n* = 24) [group definition]	7 (29.17) 7 (29.17)	27.58 (10.33) 27.00 (9.81)	ICD/DSM	Observational, cross-sectional (8)	Psychopathology
Skodlar and Parnas 2010^76^	Slovenia	**- Schizophrenia spectrum psychosis (*n* = 25)**	10 (40)	–	ICD-10/DSM-IV	Observational, cross-sectional (7)	Psychopathology (suicidality)
Piani 2025^41^	Switzerland	**- Schizophrenia (*n* = 15)** **- Schizoaffective disorder (*n* = 5)** **- APPD with symptoms of SCZ (*n* = 6)** - APPD without symptoms of SCZ (*n* = 4) [*n* < 5] - Substance induced psychotic disorder (*n* = 1) [*n* < 5] - Schizotypal disorder (*n* = 1) [*n* < 5] - Depression with psychotic symptoms (*n* = 1) [*n* < 5] - Psychosis risk syndrome (*n* = 2) [*n* < 5]	– – – – – – – –	– – – – – – – –	ICD-10	Observational, cross-sectional (5)	EEG task (pre-reflective and reflective self-processing)
Park 2020^77^	Republic of Korea	**- CHR-P: UHR (*n* = 19)** **- Healthy controls (*n* = 24)**	– –	– –	DSM-IV, SIPS	Observational, cross sectional—(6)	MRI task (self-referential processing, perspective-taking)
Continuous EASE scoring method							
Barata 2025^83^	Australia	**- CHR-P: UHR remission at 12-months (*n* = 18)** ^c^ **- CHR-P: UHR persistence/transition at 12-months (*n* = 25)** ^c^ - Mixed psychotic disorders: FEP (*n* = 38) [group definition]	– – –	– – –	CAARMS	Observational, longitudinal (5)	Psychopathology
Nelson 2012^11^	Australia	**- CHR-P: UHR (*n* = 49)** **- Healthy controls (*n* = 52)** ^e^	27 (55.1) 27 (51.9)	19.22 (2.90) 20.10 (2.84)	DSM-IV, CAARMS	Observational, longitudinal (8)	Psychopathology
Nelson 2019^84^	Australia	**- CHR-P: UHR (*n* = 21)** **- Healthy controls (*n* = 11)** - Mixed psychotic disorders: FEP (*n* = 14) [group definition]	– – –	– – –	CAARMS	Observational, cross sectional (5)	Psychopathology (psychometric)
Nelson 2020^56^	Australia	**- CHR-P: UHR (*n* = 50)** - Healthy controls (*n* = 34) [overlapping] - Mixed psychotic disorders: FEP (*n* = 39) [group definition]	28 (56) 24 (71) 21 (54)	18.78 (4.93) 21.09 (1.85) 19.87 (3.25)	CAARMS	Observational, cross sectional (7)	Psychopathology, neurocognition, and EEG task (source monitoring, aberrant salience)
Rasmussen 2020^57^	Australia	**- Schizophrenia spectrum psychosis (*n* = 26)** - CHR-P: UHR (*n* = 38) [overlapping] - Healthy controls (*n* = 33) [overlapping]	15 (57.69) 23 (50.63) 24 (72.73)	19.9 (2.8) 19.4 (2.8) 21.1 (1.9)	DSM-IV, CAARMS	Observational, cross sectional (8)	Psychopathology
Spark 2021^58^	Australia	**- Non-schizophrenia spectrum psychosis (*n* = 21)** **- Healthy controls (*n* = 34)** - Schizophrenia spectrum psychosis (*n* = 16) [overlapping]	– 25 (73.5) –	– 21.09 (1.85) –	DSM-IV	Observational, cross-sectional (7)	Psychopathology and neurocognition
Comparelli 2016^68^	Italy	**- CHR-P: UHR (*n* = 45)** - Mixed clinical sample (*n* = 70) [group definition]	23 (51.1) 28 (40)	21.04 (0.4) 20.63 (0.3)	SIPS, DSM-IV	Observational, cross-sectional (7)	Psychopathology and neurocognition
Donati 2021^71^	Italy	**- Schizophrenia spectrum psychosis (*n* = 10)**	1 (10)	25.3 (4.0)	DSM-5	Observational, cross-sectional (4)	EEG task (brisk fist closure)
Binary and continuous EASE scoring method							
Nelson 2013^55^	Australia	**- Schizophrenia spectrum psychosis (*n* = 8)** **- Non-schizophrenia spectrum psychosis (*n* = 8)**	4 (50) 2 (25)	22.25 (4.23) 21.00 (3.30)	DSM-IV	Observational, cross-sectional (7)	Psychopathology
Baklund 2024^36^	Norway	**- CHR-P: UHR (*n* = 27)**	16 (59.3)	16.1 (1.2)	SIPS	Observational, longitudinal (7)	Psychopathology
Værnes 2019^73^	Norway	**- CHR-P: UHR (*n* = 31) [Schizotypal disorder (*n* = 6)** ^f^ **]** - Non-progressive APS (*n* = 7) [group definition]	13 (41.94) 1 (14.29)	19 (3.3) 23.1 (3.7)	DSM-IV, SIPS	Observational, cross-sectional (8)	Psychopathology

Samples in bold were included in the meta-analytic estimates.

Abbreviations: APS = attenuated psychotic symptoms; APPD = acute polymorphic psychotic disorder; CAARMS = Comprehensive Assessment of At Risk Mental States; CHR-P = clinical high-risk for psychosis; COPER = Cognitive-Perceptive basic symptoms criteria; COGDIS = Cognitive basic symptoms criteria; DSM = Diagnostic and Statistical Manual of Mental Disorders; EASE = Examination of Anomalous Self-Experiences; EEG = electroencephalography; Exp. = experimental study; FEP = first episode of psychosis; ICD = International Classification of Diseases; MD = major depression, fMRI = functional magnetic resonance imaging; NOS = not otherwise specified by the authors; OCD = obsessive-compulsive disorder; SCZ = schizophrenia; SD = standard deviation; SIPS = Structured Interview for Psychosis-Risk Syndromes; SPD = schizotypal personality disorder; SPI-A/CY = Schizophrenia Proneness Instrument, Adult (SPI-A) and Child Youth (SPI-CY) versions; UHR = ultra-high-risk for psychosis. Data not available/found are indicated with a dot.

^a^Only included in major diagnostic categories analyses due to overlap.

^b^Not included in major diagnostic categories analyses due to overlap.

^c^Only EASE dimensions scores used in the analyses.

^d^Mean and standard deviation of EASE scores obtained from Madeira et al. commentary report,^108^ as the original publication reports EASE scores including sub-items.

^e^Includes 2 controls with a diagnosis of affective disorders and 2 with other (non-affective and non-anxiety) Axis-I disorder.

^f^Subsample of CHR-P individuals with a diagnosis of schizotypal disorder only included in the binary EASE scores meta-analysis.

### EASE Scores Across Mental Disorders, Risk Syndromes, and Healthy Controls

#### Binary EASE Scores

Forty-four samples (*n* = 858 patients, *n* = 130 healthy controls) were included in the meta-analytic synthesis for EASE binary scores (Table 2). See Supplementary material for list of studies included in each binary estimate (Table S7). The meta-analytic total EASE score was 19.12 in schizotypal disorder (*k* = 6, *n* = 94), 17.81 in schizophrenia spectrum psychosis (*k* = 14, *n* = 334), 13.89 in CHR-P individuals (*k* = 10, *n* = 230), 8.64 in non-schizophrenia spectrum psychosis (*k* = 4, *n* = 48), and 0.84 in healthy controls (*k* = 4, *n* = 130). In addition, single studies reported binary total EASE scores for borderline personality disorder (14.71, *n* = 27), panic disorder (13.11, *n* = 47), Asperger’s syndrome (7.36, *n* = 22), affective disorders (5.70, *n* = 9), and OCD (5.33, *n* = 12). Heterogeneity (*I*^2^ index) for total binary EASE scores was moderate to high (from 46.7% to 89.1%).

**Table 2:** Weighted Means of Binary EASE Scores Across Mental Disorders, Risk-syndromes, and Healthy Controls

EASE domains	Schizophrenia spectrum psychosis			**Non-SCZS psychosis** ^**a**^		
	*k* (*n*)	Mean (95% CI)	***I*** ^**2**^	*k* (*n*)	Mean (95% CI)/[SD]	***I*** ^**2**^
Total EASE score	14 (334)	17.81 (15.17-20.45)	89.1	4 (48)	8.64 (5.90-11.38)	46.7
1. Cognition and stream of consciousness	9 (236)	6.94 (5.98-7.89)	79.1	1 (8)	4.5 [3.21]	–
2. Self-awareness and presence	7 (203)	7.26 (6.09-8.44)	82.3	1 (8)	3.25 [2.60]	–
3. Bodily experiences	7 (203)	2.03 (1.50-2.56)	77.2	1 (8)	0.34 [7.44]	–
4. Demarcation/transitivism	6 (178)	1.11 (0.73-1.50)	80.9	1 (8)	0.38 [0.52]	–
5. Existential reorientation	6 (178)	2.18 (1.69-2.67)	68.5	1 (8)	1.75 [1.67]	–
**EASE domains**	**Schizotypal disorder**			**Borderline personality disorder**		
	***k* (*n*)**	**Mean (95% CI)/[SD]**	***I*** ^**2**^	***k* (*n*)**	**Mean [SD]**	***I*** ^**2**^
Total EASE score	6 (94)	19.12 (15.97-22.27)	87.9	1 (27)	14.71 [7.36]	–
1. Cognition and stream of consciousness	1 (29)	9.72 [2.63]	–	–	–	–
2. Self-awareness and presence	1 (29)	9.03 [2.28]	–	–	–	–
3. Bodily experiences	1 (29)	2.24 [1.64]	–	–	–	–
4. Demarcation/transitivism	1 (29)	0.97 [0.87]	–	–	–	–
5. Existential reorientation	1 (29)	3.28 [1.87]	–	–	–	–
**EASE domains**	**Asperger’s syndrome**			**Affective disorders**		
	***k* (*n*)**	**Mean [SD]**	***I*** ^**2**^	***k* (*n*)**	**Mean [SD]**	***I*** ^**2**^
Total EASE score	1 (22)	7.36 [3.49]	–	1 (9)	5.70 [7.10]	–
1. Cognition and stream of consciousness	1 (22)	4.36 [1.84]	–	–	–	–
2. Self-awareness and presence	1 (22)	2.05 [1.5]	–	–	–	–
3. Bodily experiences	1 (22)	0.27 [0.46]	–	–	–	–
4. Demarcation/transitivism	1 (22)	0.23 [0.43]	–	–	–	–
5. Existential reorientation	1 (22)	0.45 [0.74]	–	–	–	–
**EASE domains**	**Panic disorder**			**Obsessive-compulsive disorder**		
	***k* (*n*)**	**Mean [SD]**	***I*** ^**2**^	***k* (*n*)**	**Mean [SD]**	***I*** ^**2**^
Total EASE score	1 (47)	13.11 [8.80]	–	1 (12)	5.33 [3.47]	–
1. Cognition and stream of consciousness	–	–	–	–	–	–
2. Self-awareness and presence	–	–	–	–	–	–
3. Bodily experiences	–	–	–	–	–	–
4. Demarcation/transitivism	–	–	–	–	–	–
5. Existential reorientation	–	–	–	–	–	–
**EASE domains**	**CHR-P**			**Healthy controls**		
	***k* (*n*)**	**Mean (95% CI)**	***I*** ^**2**^	***k* (*n*)**	**Mean (95% CI)/[SD]**	***I*** ^**2**^
Total EASE score	10 (230)	13.89 (11.35-16.42)	88.6	4 (130)	0.84 (0.55-1.13)	70.8
1. Cognition and stream of consciousness	2 (34)	3.97 (−0.6 to 8.54)	96.5	1 (35)	0.31 [0.63]	–
2. Self-awareness and presence	2 (34)	2.95 (1.09-4.81)	82.3	1 (35)	0.34 [0.68]	–
3. Bodily experiences	2 (34)	1 (0.48-1.52)	0	1 (35)	0.03 [0.17]	–
4. Demarcation/transitivism	2 (34)	0.29 (0.08-0.5)	35.7	1 (35)	0 [0]	–
5. Existential reorientation	2 (34)	0.48 (−0.13 to 1.08)	77.6	1 (35)	0.26 [0.44]	–

^a^Includes delusional disorder or psychosis not otherwise specified (*n* = 18), bipolar disorder with psychotic features (*n* = 22), acute polymorphic psychotic disorder with symptoms of schizophrenia (*n* = 6), affective disorder with psychotic features (*n* = 1), and substance-induced psychotic disorder (*n* = 1).

Domain-specific binary EASE scores were only available for a subsample of the disorders, with most of the estimates relying in single studies or small samples of ≤35 participants (Table 2). Schizophrenia spectrum disorders had the highest number of available studies and participants (*k* = 9-6, *n* = 236-178) with domain-specific scores being higher for domains 1 and 2 (mean scores of 6.94 and 7.26, respectively).

#### Continuous EASE Scores

Fifteen samples (*n* = 318 patients, *n* = 97 healthy controls) were included in the meta-analytic synthesis for EASE continuous scores (Table 3). See Supplementary material for list of studies included in each continuous estimate (Table S8). The meta-analytic total EASE score was 73.63 in schizophrenia spectrum psychosis (*k* = 3, *n* = 44), 51.05 in CHR-P individuals (*k* = 5, *n* = 202), 47.32 in non-schizophrenia spectrum psychosis (*k* = 2, *n* = 29), and 4.33 in healthy controls (*k* = 3, *n* = 97). There were not enough data to conduct a meta-analysis for the other disorders in the continuous estimates. Heterogeneity (*I*^2^ index) for total continuous EASE scores was high for all groups (from 63.3% to 90.2%), except for schizophrenia spectrum psychosis (0%).

**Table 3:** Weighted Means of Continuous EASE Scores (Cumulative Frequency and Severity) Across Mental Disorders, Risk-Syndromes, and Healthy Controls

	Schizophrenia spectrum psychosis			**Non-SCZS psychosis** ^**a**^		
	*k* (*n*)	Mean (95% CI)	***I*** ^**2**^	*k* (*n*)	Mean (95% CI)	***I*** ^**2**^
Total EASE score	3 (44)	73.63 (63.25-84.02)	0	2 (29)	47.32 (20.03-74.05)	90.2
1. Cognition and stream of consciousness	2 (18)	25.41 (18.73-32.09)	0	2 (29)	20.56 (7.90-33.22)	93.2
2. Self-awareness and presence	2 (18)	28.92 (22.44-35.41)	0	2 (29)	14.60 (7.65-21.56)	75.0
3. Bodily experiences	2 (18)	6.77 (3.68-9.87)	0	2 (29)	3.89 (−1.11 to 8.89)	91.1
4. Demarcation/transitivism	2 (18)	4.17 (2.67-5.66)	0	2 (29)	1.89 (0.09-3.69)	72.2
5. Existential reorientation	2 (18)	10.51 (7.75-13.26)	0	2 (29)	6.22 (4.47-7.98)	0
**EASE domains**	**CHR-P**			**Healthy controls**		
	***k* (*n*)**	**Mean (95% CI)**	***I*** ^**2**^	***k* (*n*)**	**Mean (95% CI)**	***I*** ^**2**^
Total EASE score	5 (202)	51.05 (45.05-57.05)	63.3	3 (97)	4.33 (1.86-6.79)	83.9
1. Cognition and stream of consciousness	4 (137)	22.65 (16.86-28.45)	88.2	–	–	–
2. Self-awareness and presence	4 (137)	19.37 (13.67-25.08)	89.3	–	–	–
3. Bodily experiences	4 (137)	3.43 (1.4-5.46)	92.3	–	–	–
4. Demarcation/transitivism	4 (137)	1.88 (0.7-3.06)	89.6	–	–	–
5. Existential reorientation	4 (137)	3.59 (2.5-4.67)	38.7	–	–	–

Abbreviations: CHR-P = clinical high-risk state for psychosis; CI = confidence interval; *k* = number of samples; *n* = number of participants; SCZS = schizophrenia spectrum.

^a^Includes delusional disorder or psychosis not otherwise specified (*n* = 17), bipolar disorder with psychotic features (*n* = 1), affective disorder with psychotic features (*n* = 10), and substance-induced psychotic disorder (*n* = 1).

Domain-specific continuous EASE scores were only available for schizophrenia spectrum psychosis (*k* = 2, *n* = 18), non-schizophrenia spectrum psychosis (*k* = 2, *n* = 29) and CHR-P individuals (*k* = 4, *n* = 137, Table 3). For all 3 clinical groups, the highest scores were observed in domain 1 (mean scores of 20.56-25.41) and domain 2 (14.60-28.92).

### Meta-Regression Analyses and Pairwise Comparisons

**Figure 2:**
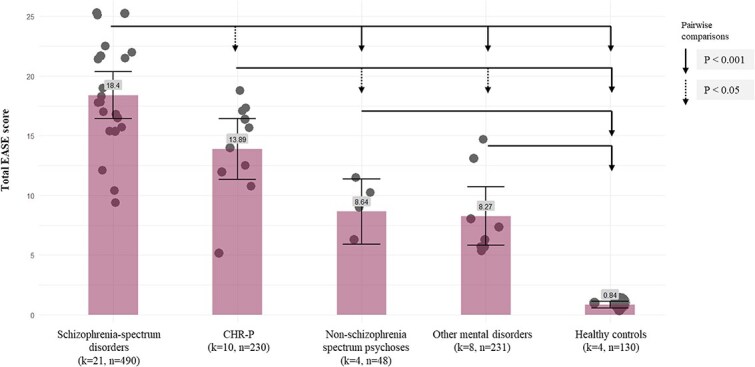
Pairwise Comparisons of Total EASE (Binary) Scores Across Schizophrenia-Spectrum Disorders, Non-schizophrenia Spectrum Psychotic Disorders, Other Mental Disorders, Clinical High-risk for Psychosis (CHR-P), and Healthy Controls—All Eligible Studies.

Sensitivity analyses of total EASE binary scores from all eligible studies was based on 47 distinct samples grouped across 5 major diagnostic categories (Figure 2, Table S9). See Supplementary material for forest plot (Figure S1). We obtained a pooled mean score of 18.40 for schizophrenia spectrum disorders (*k* = 21, *n* = 490), 13.89 for CHR-P individuals (*k* = 10, *n* = 230), 8.64 for non-schizophrenia spectrum psychosis (*k* = 4, *n* = 48), 8.27 for other mental disorders (*k* = 8, *n* = 231), and 0.84 for healthy controls (*k* = 4, *n* = 130). The major diagnostic categories variable accounted for 70.3% of the observed between-study heterogeneity in EASE scores (QM(4) = 98.64, *P* < .001, *R*^2^ = 0.703, Figure 2). In pairwise comparisons (Figure 2, Table S10), schizophrenia spectrum disorders (*k* = 21) showed significantly higher total EASE binary scores than all other major diagnostic categories: vs healthy controls (*k* = 4, *Q* = 68.25, *P* < .001), vs other mental disorders (*k* = 8, *Q* = 33.33, *P* < .001), vs non-schizophrenia spectrum psychosis (*k* = 4, *Q* = 14.28, *P* < .001), and vs CHR-P individuals (*k* = 10, *Q* = 7.14, *P* = .007). All major clinical diagnostic categories presented significantly higher total EASE scores when compared to healthy controls: non-schizophrenia spectrum psychosis vs healthy controls (*Q* = 60.85, *P* < .001), CHR-P vs healthy controls (*Q* = 46.08, *P* < .001), and other mental disorders vs healthy controls (*Q* = 20.80, *P* < .001). CHR-P presented higher total EASE scores than other mental disorders (*Q* = 9.45, *P* = .002) and non-schizophrenia spectrum psychosis (*Q* = 4.20, *P* = .041). There were no significant differences in non-schizophrenia spectrum psychosis vs other mental disorders (*Q* = 0.10, *P* = .754).

When the analyses were restricted to a selection of 30 high-methodological quality samples, the major diagnostic categories accounted for 90.18% of the observed between-study heterogeneity in total EASE binary scores (QM(4) = 218.965, *P* < .001, *R*^2^ = 0.902). See Supplementary material for forest plot (Figure S2). In pairwise comparisons, differences between major diagnostic categories remained significant at the *P* < .001 level, with generally more accentuated group differences and reduced within-group heterogeneity (Tables S11 and S12, Figure S3). The only exceptions were CHR-P vs non-schizophrenia spectrum psychosis (*P* = .006) and non-schizophrenia spectrum psychosis vs other mental disorders (*P* = .491).

An exploratory meta-regression restricted to schizophrenia-spectrum samples did not detect a statistically significant effect of study region (Denmark vs non-Denmark) on total binary EASE scores (QM(1) = 3.13, *P* = .077), accounting for 11.1% of heterogeneity, while residual heterogeneity remained high (*I*^2^ = 87.5%) (Figure S4).

## Discussion

This evidence synthesis examined the distribution of BSD across a wide range of mental disorders, CHR-P individuals, and healthy controls. To our knowledge, this represents the most comprehensive quantitative synthesis of EASE-based BSD research to date. Compared to previous reviews,^13,17,28-30^ this study applied homogeneous assessment methods based on the EASE, examined both total and domain-specific BSD using binary and continuous scoring methods, and included CHR-P individuals.

Our synthesis indicates that EASE-defined BSD have been reported across different types of mental disorders. At the same time, mean total EASE scores were markedly higher in schizophrenia and related psychoses (mean total score of 17.81) and in schizotypal disorder (mean total score of 19.12) than in other diagnostic groups, including non-schizophrenia spectrum psychotic disorders, affective disorders, panic disorder, OCD, borderline personality disorder, and Asperger’s syndrome. In meta-regressions, the major diagnostic categories explained 70.3% of the observed between-study heterogeneity in EASE scores (90.18% if only high-quality samples were included).

Notably, two studies reported EASE scores of ≥11 for borderline personality disorder^38^ and panic disorder,^22^ above the suggested specificity threshold for schizophrenia spectrum diagnosis.^15^ These findings raise important questions regarding the specificity of BSD for schizophrenia spectrum, as originally suggested by the IDM.

Several non-mutually exclusive interpretations may account for the elevated EASE scores reported in non-schizophrenia spectrum samples. One possibility is that such scores might reflect unrecognized schizophrenia spectrum vulnerability in clinical samples, underscoring the importance of comprehensive psychopathological assessments, including the screening of schizotypal traits and CHR-P status. In this regard, methodological features of these studies warrant consideration. The panic disorder sample consisted of patients with severe, treatment-nonresponsive presentations and relied on diagnostic procedures that did not systematically assess schizotypal traits or CHR-P status, thereby limiting interpretability.^22^ Similarly, the borderline personality disorder sample was characterized by high rates of comorbidity, complicating its evaluation as a distinct clinical group.^38^

A second, complementary interpretation, drawn from recent phenomenological theory, concerns the theoretical distinction between primary and secondary forms of anomalous self-experience.^27,85^ From this perspective, BSD may arise either as automatic, passively experienced alterations basic selfhood, as well as secondary, reactive, or compensatory responses to environmental stressors, trauma, or affective overload.^27^ Some secondary forms of basic-self disturbances may also be volitionally initiated, as observed in some forms of hyper-reflexivity in the context of intense introspectionism.^86^ Importantly, this primary-secondary distinction remains largely conceptual and requires further empirical validation.

Both primary and secondary processes have been proposed as potential contributors to BSD expression in schizophrenia, suggesting that the trait-like nature of BSD does not imply phenomenological invariance or state-independence.^27^ From a developmental perspective, longitudinal studies have shown that elevated levels of BSD can precede the onset of schizophrenia spectrum psychoses^87^ and may remain relatively stable over time,^88,89^ consistent with their role as early vulnerability markers. This pattern aligns with our findings of elevated EASE scores in schizotypal disorder and in individuals at clinical high risk for psychosis (CHR-P). This early vulnerability has been suggested to interact with metacognitive,^90^ cognitive,^91-93^ and neurocognitive processes,^94,95^ as well as environmental stressors,^27^ contributing to secondary self-disturbances. In line with this, emerging empirical evidence indicates that anomalous self-experiences and cognitive biases may mediate the relationship between trauma exposure, personality traits, and psychosis risk in non-clinical samples,^91-93^ pointing to mechanisms that might operate dimensionally across levels of clinical severity.

In this context, BSD phenomena observed in non-schizophrenia spectrum conditions may be interpreted as reflecting secondary or reactive forms of self-disturbance, often overlapping with depersonalization and derealization experiences, rather than the primary disturbance in self-experience observed in schizophrenia.^96^ If so, this would imply that while some items of the EASE can occur across diagnostic categories, their developmental origin, phenomenology, and clinical significance could differ substantially. This would also indicate that some items in the EASE can be observed across diagnoses, being less specific to schizophrenia-spectrum conditions as originally proposed in phenomenological accounts.

More generally, however, these considerations remain primarily theoretical and require further validation by methodological homogeneous empirical studies. Notably, the available evidence remains limited by small samples (*n* ≤ 50) for panic disorder, OCD, borderline personality disorder, Asperger’s syndrome, and psychotic disorders outside of the schizophrenia spectrum. Domain-specific scores, which could point to the specificity of some EASE domains (eg, bodily experiences) among non-schizophrenia spectrum conditions, were often not reported. Moreover, the cross-sectional nature of most studies does not allow the examination of diagnostic changes over time. For example, a 5-years follow-up study of 121 first-admission patients found that elevated levels of BSD, measured via a proto-EASE scale, predicted a re-diagnosis with a schizophrenia spectrum condition at follow-up.^97^ Accordingly, external validation of BSD outside schizophrenia spectrum and CHR-P populations remains limited, as few studies have examined their longitudinal associations with clinically meaningful outcomes.

Taken together, current evidence suggests a graded distribution of EASE-defined BSD across diagnostic groups, with a marked hyperaggregation in schizophrenia spectrum conditions. Whether the self-disturbances observed outside of the schizophrenia spectrum are best conceptualized as secondary or reactive phenomena remains to be elucidated.

On a separate note, these findings can inform the diagnostic refinement and distinction between schizophrenia and non-schizophrenia spectrum conditions. In line with previous reviews,^13,17,29^ our findings are compatible with the hypothesis that BSD might capture an important phenomenological dimension of schizophrenia and related conditions.^7,98^ Moreover, ICD/DSM diagnostic categories represent polythetic clinical constructs that aggregate heterogeneous symptom profiles and mechanisms.^99,100^ From this perspective, the comparisons of EASE-defined BSD across diagnostic groups may partly reflect variability within diagnostic categories, rather than purely between them. At the same time, the presence of BSD-like phenomena outside the schizophrenia spectrum suggests that further refinement of the EASE might be required to distinguishing putative primary and secondary forms of BSD and improve feasibility for real-world clinical use. Future longitudinal research combining phenomenological and translational approaches will be needed to clarify the role of BSD across diagnostic boundaries and inform the refinement of current diagnostic frameworks.

Interestingly, we also found higher levels of BSD in CHR-P individuals, compared to non-schizophrenia spectrum psychotic disorders, other mental disorders, and healthy controls. This intermediate pattern of EASE scores in CHR-P samples was observed in both binary and continuous estimates, indicating convergence between presence-only and presence-severity-based assessments in this risk group. This finding holds relevant preventive potential as high levels of BSD may predict symptom persistence^83^ and psychosis onset among CHR-P individuals and non-psychotic help-seeking adolescents.^11,83,87^ This is particularly relevant given the substantial comorbidity and clinical heterogeneity of the CHR-P state and the need to improve risk-stratification of CHR-P individuals.^101^ Existing CHR-P criteria only partially capture BSD, and their future refinement could consider a more in-depth assessment of this dimension. Information on BSD in CHR-P individuals could be combined with other genetic and non-genetic risk and protective factors^102^ to improve prognostic accuracy of current assessment tools and identify individuals with predominantly affective or anxious symptomatology.^44^

As an observational note, despite not being a primary focus of the search strategy, we did not identify studies assessing the effects of biological or psychosocial interventions on EASE scores. This gap is noteworthy, as BSD represent a largely unexplored domain for the clinical management of psychotic disorders. EASE scores have been associated with a wide range of clinical outcomes in CHR-P or individuals with psychosis, including symptom severity and functioning, as measured by the Global Assessment of Functioning scale,^68,69,88,103,104^ role^70^ and social^105^ functioning, personality functioning,^38^ socio-professional adjustment,^60^ self-harm,^57^ suicidality,^76,81^ and symptomatic recovery.^106^ Guideline-recommended^107^ cognitive-behavioral therapy interventions have been suggested to be of limited impact for BSD,^108,109^ and some types of psychotherapy for schizophrenia can be informed by the basic-self concept.^110-112^ However, none of these approaches have utilized the EASE, or alternative instruments, as outcome measures. Importantly, given the suggested trait-like nature of at least some BSD, the primary objective of interventions would not necessarily be to “cure” these experiences.^110^ Rather, phenomenological principles may be embedded within psychotherapeutic approaches to enhance insight into BSD and their consequences on patients’ lives, alleviating the associated distress, and support recovery and functioning.^110,113^ From this perspective, therapeutic efforts would be more appropriately evaluated in terms of changes in distress, social functioning, or well-being rather than reductions in self-disorders per se. In any case, it remains unclear to what extent BSD can be modified with repurposed or novel interventions, and whether such changes would translate into meaningful clinical outcomes. In the future, new abbreviated versions of the EASE,^114^ or other cost-saving alternatives for the assessment of BSD,^14^ could facilitate interventional studies on BSD.

The results of the meta-analysis should be interpreted considering the methodological limitations of our analyses and of EASE research more broadly. Overall methodological quality was moderate to high (median = 7), with 84.2% of studies obtaining a score of ≥6 over a maximum of 10. This is expected since we only included studies on samples that had undergone a clinical assessment process for their selection (eg, studies relying on self-rating scales were excluded), and which used the gold-standard instrument for the assessment of BSD. We intentionally excluded studies using self-report measures or alternative instruments with less established validity.^14^

Nonetheless, only 15.8% of studies explicitly specified the timeframe used to assess BSD. The EASE allows for either lifetime assessment of BSD (ie, since childhood until the time of assessment) or restricted time windows (eg, the last 2 weeks).^8^ While it could be assumed that, by default, researchers are assessing lifetime presence of BSD, this is generally not explicitly reported in publications. Differences in assessment timeframe may therefore represent a source of confounding, particularly if lifetime assessments were more frequently applied in schizophrenia spectrum samples. Consequently, future EASE researchers should explicitly clarify the temporal frame employed in EASE interviews to improve comparability across studies.

Another potential source of between-study variability relates to rater expertise and regional diagnostic traditions. Several included studies involved researchers with direct or indirect links to the Copenhagen research group that originally developed IDM and the EASE. While this may enhance assessment reliability, it also introduces the possibility that EASE scores are influenced by contextual factors, such as interviewer expertise or local diagnostic conventions. Moreover, most (76.3%) studies were conducted in a limited number of countries (Denmark, Australia, Italy or Norway), indicating a relative regional concentration of EASE research. An exploratory meta-regression restricted to schizophrenia-spectrum samples did not detect a statistically reliable effect of study region (Denmark vs non-Denmark) on total binary EASE scores, although residual heterogeneity remained high and the analysis was underpowered. As the EASE literature expands, future studies will be better positioned to examine whether systematic regional patterns in scores exist, or whether the distribution of EASE scores remains robust across different cultural and clinical settings.

Several meta-analytic estimates were also based on relatively small sample sizes (*n* < 30) or number of included studies (*k* ≤ 5), and should therefore be interpreted cautiously.^115^ For panic disorder (*n* = 47), borderline personality disorder (*n* = 27), Asperger’s syndrome (*n* = 22), OCD (*n* = 12) and affective disorders (*n* = 9), only single studies were available, so no meta-analytic syntheses were conducted. Although statistical power was improved in our pairwise comparisons by aggregating studies into major diagnostic categories, this could not be replicated for analyses at level of EASE domains.

Consistent with these constraints, heterogeneity was moderate to high in many estimates. The residual heterogeneity of almost 30% observed in meta-regression analyses likely reflects the combined influence of unreported EASE assessment timeframes, regional diagnostic traditions, variability in interviewer expertise, and differences in sample characteristics, which could not be formally modeled given current data limitations. Interrater reliability estimates based on recorded interviews may also not fully generalize to live assessments, particularly given the importance of interviewer expertise in the administration of the EASE. Although heterogeneity was reduced when estimated were limited to the higher-quality samples, it remained moderate to high. Finally, the observed low heterogeneity in some estimates, most notably for continuous EASE scores in schizophrenia-spectrum psychosis (*I*^2^ = 0, *k* = 3), should be interpreted cautiously, as it might reflect the small number of included studies and the resulting limited power to detect between-study variance.

## Conclusion

The available evidence indicates that EASE-defined BSD are detectable across multiple psychiatric categories but show a graded distribution with markedly higher levels in schizophrenia spectrum conditions, and are also prominently expressed in CHR-P individuals. Further longitudinal and methodologically harmonized research is needed to clarify the trajectories, clinical significance, and prognostic implications of BSD across diagnostic categories.

## Supplementary Material

sbag047_Supplemental_File
