# Supplementary material for: Basic Self-disorders Across Psychiatric Diagnoses and Risk Syndromes: An Updated Meta-analysis
Source: Schizophr Bull. 2026 May 11;52(3):sbag047. doi: 10.1093/schbul/sbag047 (PMC13158234; doi:10.1093/schbul/sbag047)
Supplement: sbag047_Supplemental_File [file sbag047_supplemental_file.docx]

**SUPPLEMENTARY MATERIAL**

Contents

[sTable 1: PRISMA statement and checklist 2](#_Toc220783839)

[sTable 2: MOOSE Statement – Reporting Checklist for Authors, Editors, and Reviewers of Meta-analyses of Observational Studies 4](#_Toc220783840)

[sTable 3. TRANSD criteria employed for the meta-analysis^1,2^ 6](#_Toc220783841)

[sTable 4. Methodological quality and risk of bias tool for studies included in the meta-analysis 7](#_Toc220783842)

[sTable 5. Studies investigating basic self-disorders using the EASE excluded from the meta-analysis 10](#_Toc220783843)

[sTable 6. Quality analysis 13](#_Toc220783844)

[sTable 7. Studies included in estimates of weighted means of binary EASE scores 16](#_Toc220783845)

[sTable 8. Studies included in estimates of weighted means of continuous EASE scores 18](#_Toc220783846)

[sTable 9. EASE total scores (binary) by major diagnostic categories - all eligible samples 19](#_Toc220783847)

[sTable 10. Pairwise comparisons of EASE total scores (binary) by major diagnostic categories – all eligible samples 20](#_Toc220783848)

[sTable 11. EASE total scores (binary) by major diagnostic categories – high-quality samples 21](#_Toc220783849)

[sTable 12. Pairwise comparisons of EASE total scores (binary) by major diagnostic categories – high-quality samples 22](#_Toc220783850)

[sFigure 1. Forest plot: weighted means of total binary EASE scores across schizophrenia-spectrum disorders, non-schizophrenia spectrum psychotic disorders, other mental disorders, clinical high-risk for psychosis (CHR-P), and healthy controls – all eligible studies 23](#_Toc220783851)

[sFigure 2. Forest plot: weighted means of total binary EASE scores across schizophrenia-spectrum disorders, non-schizophrenia spectrum psychotic disorders, other mental disorders, clinical high-risk for psychosis (CHR-P), and healthy controls – high-quality samples 24](#_Toc220783852)

[sFigure 3. Pairwise comparisons of total EASE (binary) scores across schizophrenia-spectrum disorders, non-schizophrenia spectrum psychotic disorders, other mental disorders, clinical high-risk for psychosis (CHR-P), and healthy controls – high-quality samples 25](#_Toc220783853)

[sFigure 4. Forest plot of total binary EASE scores in schizophrenia-spectrum samples, stratified by study region (Denmark vs non-Denmark), used in the exploratory regional meta-regression 26](#_Toc220783854)

[References 27](#_Toc220783855)

# sTable 1: PRISMA statement and checklist

| **Section/topic** | **#** | **Checklist item** | **Page** |
| --- | --- | --- | --- |
| **TITLE** |  |  |  |
| Title | 1 | Identify the report as a systematic review, meta-analysis, or both. | Yes (title page) |
| **ABSTRACT** |  |  |  |
| Structured summary | 2 | Provide a structured summary including, as applicable: background; objectives; data sources; study eligibility criteria, participants, and interventions; study appraisal and synthesis methods; results; limitations; conclusions and implications of key findings; systematic review registration number. | 2 |
| **INTRODUCTION** |  |  |  |
| Rationale | 3 | Describe the rationale for the review in the context of what is already known. | 4 |
| Objectives | 4 | Provide an explicit statement of questions being addressed with reference to participants, interventions, comparisons, outcomes, and study design (PICOS). | 5-6 |
| **METHODS** |  |  |  |
| Protocol and registration | 5 | Indicate if a review protocol exists, if and where it can be accessed (e.g., Web address), and, if available, provide registration information including registration number. | No pre-registered protocol |
| Eligibility criteria | 6 | Specify study characteristics (e.g., PICOS, length of follow-up) and report characteristics (e.g., years considered, language, publication status) used as criteria for eligibility, giving rationale. | 5-6 |
| Information sources | 7 | Describe all information sources (e.g., databases with dates of coverage, contact with study authors to identify additional studies) in the search and date last searched. | 5 |
| Search | 8 | Present full electronic search strategy for at least one database, including any limits used, such that it could be repeated. | 5 |
| Study selection | 9 | State the process for selecting studies (i.e., screening, eligibility, included in systematic review, and, if applicable, included in the meta-analysis). | 6 |
| Data collection process | 10 | Describe method of data extraction from reports (e.g., piloted forms, independently, in duplicate) and any processes for obtaining and confirming data from investigators. | 6 |
| Data items | 11 | List and define all variables for which data were sought (e.g., PICOS, funding sources) and any assumptions and simplifications made. | 6 |
| Risk of bias in individual studies | 12 | Describe methods used for assessing risk of bias of individual studies (including specification of whether this was done at the study or outcome level), and how this information is to be used in any data synthesis. | 8, Supplementary |
| Summary measures | 13 | State the principal summary measures. | 7 |
| Risk of bias across studies | 15 | Specify any assessment of risk of bias (i.e. Newcastle-Ottawa Scale (NOS), that may affect the cumulative evidence. | 8 |
| Additional analyses | 16 | Describe methods of additional analyses (e.g., sensitivity or subgroup analyses, meta-regression), if done, indicating which were pre-specified. | 7-8 |
| **RESULTS** |  |  |  |
| Study selection | 17 | Give numbers of studies screened, assessed for eligibility, and included in the review, with reasons for exclusions at each stage, ideally with a flow diagram. | Figure 1 |
| Study characteristics | 18 | For each study, present characteristics for which data were extracted (e.g., study size, PICOS, follow-up period) and provide the citations. | Table 1 |
| Risk of bias within studies | 19 | Present data on risk of bias of each study and, if available, any outcome level assessment (see item 12) | Supplementary |
| Results of individual studies | 20 | For all outcomes considered (benefits or harms), present, for each study a summary data for each intervention group | Supplementary |
| Synthesis of results | 21 | Present results of study analyzed. | 8-11, Tables 2-3, Figure 2, Supplementary |
| Risk of bias across studies | 22 | Present results of any assessment of risk of bias across studies (see Item 15). | Table 1, Supplementary |
| Additional analysis | 23 | Give results of additional analyses, if done (e.g., sensitivity or subgroup analyses, meta-regression [see Item 16]). | 8-11 |
| **DISCUSSION** |  |  |  |
| Summary of evidence | 24 | Summarize the main findings including the strength of evidence for each main outcome; consider their relevance to key groups (e.g., healthcare providers, users, and policy makers). | 11-15 |
| Limitations | 25 | Discuss limitations at study and outcome level (e.g., risk of bias), and at review-level (e.g., incomplete retrieval of identified research, reporting bias). | 15-17 |
| Conclusions | 26 | Provide a general interpretation of the results in the context of other evidence, and implications for future research. | 17 |
| **FUNDING** |  |  |  |
| Funding | 27 | Describe sources of funding for the systematic review and other support (e.g., supply of data); role of funders for the systematic review. | 17 |

# sTable 2: MOOSE Statement – Reporting Checklist for Authors, Editors, and Reviewers of Meta-analyses of Observational Studies

| **Reporting Criteria** | **Reported (Yes/No)** | **Reported Page** |
| --- | --- | --- |
| **Reporting of Background** |  |  |
| Problem definition | Yes | 3-5 |
| Hypothesis statement | NA | - |
| Description of Study Outcome(s) | Yes | 6-7 |
| Type of exposure or intervention used | Yes | 6 |
| Type of study design used | Yes | 6 |
| Study population | Yes | 6 |
| **Reporting of Search Strategy** |  |  |
| Qualifications of searchers (eg, librarians and investigators) | No | - |
| Search strategy, including time period included in the synthesis and keywords | Yes | 5 |
| Effort to include all available studies, including contact with authors | No | 6 |
| Databases and registries searched | Yes | 5 |
| Search software used, name and version, including special features used (eg, explosion) | NA | - |
| Use of hand searching (eg, reference lists of obtained articles) | Yes | 6 |
| List of citations located and those excluded, including justification | Yes | Figure 1, Supplementary |
| Method for addressing articles published in languages other than English | Yes | 5 |
| Method of handling abstracts and unpublished studies | NA | - |
| Description of any contact with authors | No | - |
| **Reporting of Methods** |  |  |
| Description of relevance or appropriateness of studies assembled for assessing the hypothesis to be tested | Yes | 6 |
| Rationale for the selection and coding of data (eg, sound clinical principles or convenience) | Yes | 6 |
| Documentation of how data were classified and coded (eg, multiple raters, blinding, and interrater reliability) | Yes | 6 |
| Assessment of confounding (eg, comparability of cases and controls in studies where appropriate) | Yes | 7-8 |
| Assessment of study quality, including blinding of quality assessors; stratification or regression on possible predictors of study results | Yes | 8 |
| Assessment of heterogeneity | Yes | 7 |
| Description of statistical methods (eg, complete description of fixed or random effects models, justification of whether the chosen models account for predictors of study results, dose-response models, or cumulative meta-analysis) in sufficient detail to be replicated | Yes | 6-8 |
| Provision of appropriate tables and graphics | Yes | Figure 1, Supplementary |
| **Reporting of Results** |  |  |
| Table giving descriptive information for each study included | Yes | Table 1 |
| Results of sensitivity testing (eg, subgroup analysis) | Yes | 9-11, Tables 2-3, Figure 2, Supplementary |
| Indication of statistical uncertainty of findings | Yes | 9-11, Tables 2-3, Supplementary |
| **Reporting of Discussion** |  |  |
| Quantitative assessment of bias (eg, publication bias) | No | - |
| Justification for exclusion (eg, exclusion of non–English-language citations) | Yes | Figure 1 |
| Assessment of quality of included studies | Yes | Table 1, Supplementary |
| **Reporting of Conclusions** |  |  |
| Consideration of alternative explanations for observed results | Yes | 12-15 |
| Generalization of the conclusions (ie, appropriate for the data presented and  within the domain of the literature review) | Yes | 14, 15-17 |
| Guidelines for future research | Yes | 14-17 |

# sTable 3. TRANSD criteria employed for the meta-analysis^1,2^

| TRANSD Criterion | Assessment |
| --- | --- |
| T - Transparent definition of the gold standard (ICD, DSM, other), including specific diagnostic types, official codes, primary vs. secondary diagnoses, and diagnostic assessment interviews. | Articles selected based on DSM/ICD-any version diagnosis of any axis-I or axis-II mental disorder, ascertained clinically or with validated psychometric instruments. Risk syndromes selected based on gold-standard assessment instruments (e.g. CAARMS or SIPS). |
| R - Report the primary outcome of the study, the study design, and the definition of the transdiagnostic construct in the abstract and main text. | The primary outcome was the cumulative frequency and severity of basic-self disorders; the transdiagnostic construct is operationalised with the EASE. |
| A - Appraise the conceptual framework/approach of the transdiagnostic approach: across-diagnoses, beyond-diagnoses, other (explain). | Across diagnoses and across major diagnostic categories. |
| N - Numerate the diagnostic categories, spectra and non-clinical samples in which the transdiagnostic construct is being tested and then validated. | Major diagnostic categories and risk syndromes were explicitly defined and compared via meta-regression and subgroup analyses. |
| S - Show the degree of improvement of the transdiagnostic approach against the specific diagnostic approach through specific comparative analyses. | The present meta-analytic design does not test incremental validity or superiority of a transdiagnostic model over categorical diagnoses. |
| D - Demonstrate the generalizability of the transdiagnostic construct through external validation studies. | Associations with clinical outcomes are discussed; however, external validation outside schizophrenia spectrum and CHR-P populations remains limited in the literature. |

# sTable 4. Methodological quality and risk of bias tool for studies included in the meta-analysis

| Item | Default criteria for answers | Adapted criteria |
| --- | --- | --- |
| External validation | | |
| *1. Was the study’s target population a close representation of the national population in relation to relevant variables, e.g. age, sex, occupation?* | - **Yes (LOW RISK):** The study’s target population was a close representation of the national population. - **No (HIGH RISK)**: The study’s target population was clearly NOT representative of the national population. | The target population refers to the clinical population to which the results of the study’s results will be generalised. Rated **YES** if the clinical samples are a close representation of the national population based on key sociodemographic variables, including age, gender, occupational status, to a degree that would be expected from the same reference clinical group at a general population level. Rated **NO** if there is an overrepresentation of a specific sociodemographic group in the sample of 2/3 or more (66.6%). Also rated **NO** if a limited age group is overrepresented in the sample, or if there were several exclusion criteria that would render several potential participants ineligible. **CAN’T TELL** if sociodemographic info of sample is not available or if exclusion/exclusion criteria are not specified. |
| *2. Was the sampling frame a true or close representation of the target population?* | - **Yes (LOW RISK):** The sampling frame was a **true or close** representation of the target population. - **No (HIGH RISK)**: The sampling frame was NOT a **true or close** representation of the target population. | The **sampling frame** is a list of the sampling units in the target population and the study sample is drawn from this list. We are basing this rating on the recruitment source for the study. A **YES** indicates that the sample was recruited form a community mental health service or from general population. **NO** indicated that the sample was recruited from a specific service with a restricted catchment area of target population not representative of the community. **CAN’T TELL** if the recruitment source is not specified. |
| *3. Was some form of Random selection used to select the sample, OR, was a census undertaken?* | - **Yes (LOW RISK):** A census was undertaken, OR, some form of random selection was used to select the sample (e.g. simple random sampling, stratified random sampling, cluster sampling, systematic sampling). - **No (HIGH RISK)**: A census was NOT undertaken, AND some form of random selection was NOT used to select the sample. | Scored **YES** if there is any method for random selection of participants from a larger pool of eligible participants. We also rated as **YES** studies recruiting consecutively admitted patients. **NO/CAN’T TELL** indicates no mention of random selection of participants or consecutive admissions. |
| *4. Was the likelihood of non-response bias minimal?* | - **Yes (LOW RISK):** The response rate for the study was >/=75%, OR, an analysis was performed that showed no significant difference in relevant demographic characteristics between responders and non-responders. - **No (HIGH RISK)**: The response rate was <75%, and if any analysis comparing responders and non-responders was done, it showed a significant difference in relevant demographic characteristics between responders and non-responders. | In baseline studies, response rate is calculated as a % of all participants taking part in the study. Scored **YES** if response rate is >/=75% or if an analysis was performed that showed no significant difference in relevant demographic characteristics between responders and non-responders. |
| Internal validity | | |
| *5. Were data collected directly from the subjects (as opposed to a proxy)?* | - **Yes (LOW RISK):** All data were collected directly from the subjects. - **No (HIGH RISK):** In some instances, data were collected from a proxy. | We expect all studies to score a **YES** in this item, as this was one of the inclusion criteria – i.e. data collected via EASE scale in clinical interview. **NO** indicates the use of a proxy. **CAN’T TELL** indicates insufficient information. |
| *6. Was an acceptable case definition used in the study?* | - **Yes (LOW RISK):** An acceptable case definition was used. - **No (HIGH RISK)**: An acceptable case definition was NOT used. | While all studies used a case definition based on DSM/ICD or adequate assessment scales for risk syndromes (e.g. CAARMS/SIPS), we only consider a **YES** those studies which also assessed and screened for schizotypal traits in non-psychotic and non-CHR-P samples. Scored **NO** if SPD was not ruled out/assessed. Score **CAN’T TELL** if it’s not clearly defined how researchers obtained the ICD/DSM clinical diagnoses or if it’s not clear if SPD was ruled out. |
| *7. Was the study instrument that measured the parameter of interest (e.g. prevalence of low back pain) shown to have reliability and validity (if necessary)?* | - **Yes (LOW RISK):** The study instrument had been shown to have reliability and validity (if this was necessary), e.g. test-retest, piloting, validation in a previous study, etc. - **No (HIGH RISK)**: The study instrument had NOT been shown to have reliability or validity (if this was necessary). | Specialised training is required to utilise the EASE scale^3–5^. To score a **“YES”**, the study needs to specify that measures were employed to enhance the reliability of EASE ratings (i.e. adequate EASE training or supervision of raters, reporting of inter-rater reliability scores, or consensus meetings to discuss and review EASE ratings). |
| *8. Was the same mode of data collection used for all subjects?* | - **Yes (LOW RISK):** The same mode of data collection was used for all subjects. - **No (HIGH RISK)**: The same mode of data collection was NOT used for all subjects. | Rated **YES** if all participants underwent the same procedure for EASE data collection. |
| *9. Was the length of the shortest Prevalence period for the parameter of interest appropriate?* | - **Yes (LOW RISK):** The shortest prevalence period for the parameter of interest was appropriate (e.g. point prevalence, one-week prevalence, one-year prevalence). - **No (HIGH RISK)**: The shortest prevalence period for the parameter of interest was not appropriate (e.g. lifetime prevalence) | The prevalence period is the period that the subject is asked about e.g. “Have you experienced low back pain over the previous year?”. In this example, the prevalence period is one year. We scored **YES** if the study explicitly specifies that they are measuring lifetime basic-self disorders (since childhood). Scored **NO** if they are not measuring lifetime BSD. Scored **CAN’T TELL** if the assessment timeframe is not mentioned. |
| *10. Were the numerator(s) and denominato r(s) for the parameter of interest appropriate?* | - **Yes (LOW RISK):** The paper presented appropriate numerator(s) AND denominator(s) for the parameter of interest (e.g. the prevalence of low back pain). - **No (HIGH RISK)**: The paper did present numerator(s) AND denominator(s) for the parameter of interest but one or more of these were inappropriate. | Scored **YES** if the study reported: a) the Mean (M) and standard deviation (SD) or standard error (SE), OR b) the raw dataset of EASE scores using one of the scoring methods described in the EASE manual (binary or continuous scoring). We expect most of the studies to score a “YES”, but those which did not report the M and SD, and instead reported the M and Range, were rated as a **NO**. |

# sTable 5. Studies investigating basic self-disorders using the EASE excluded from the meta-analysis

| Study author(s) and publication year | Country | Samples | Female gender, n (%) | Mean age (SD) [range] | Group definition | Design | Reason for exclusion from meta-analysis | EASE scoring method |
| --- | --- | --- | --- | --- | --- | --- | --- | --- |
| Arnfred 2015^6^ | Denmark | - Schizophrenia (n=6) + schizotypal PD (n=6) | 7 (58.33) | 27 (5.4) | DSM-IV | Observational – EEG task | Overlapping sample | Binary |
| Handest 2025^7^ | Denmark | - Schizophrenia spectrum psychosis (n=35) | 6 (17.1) | 32.6 (.) | ICD-10 | Observational | Overlapping sample | Binary |
| Henriksen & Parnas 2012^8^ | Denmark | - Non-schizophrenia spectrum clinical sample (n=9)  - Schizophrenia spectrum psychosis (n=19)  - Schizotypal disorder (n=8) | .  .  . | .  .  . | ICD-10 | Observational | Overlapping sample | Binary |
| Nordgaard 2018^9^ | Denmark | - Non-affective psychosis and schizotypal disorder (n=48) | 30 (62.5) | 25.5 (6.33) | ICD-10 | Observational | Overlapping sample | Binary |
| Nordgaard 2019^10^ | Denmark | - Schizophrenia spectrum psychosis (n=30)  - Mixed clinical sample (n=68) | 21 (70)  44 64.7) | 27 (.)  28 (.) | ICD-10 | Observational | Group definition, overlapping sample | Binary |
| Nordgaard 2020^11^ | Denmark | - Schizophrenia spectrum psychosis (n=30)  - Mixed clinical sample (n=68) | 21 (70)  44 64.7) | 27 (.)  28 (.) | ICD-10 | Observational | Group definition, overlapping sample | Binary |
| Nordgaard 2021^12^ | Denmark | - Mixed clinical sample with bizarre responses (n=32)  - Mixed clinical sample without bizarre responses (n=62) | 21 (65.62)  42 (67.74) | 27.1 (7.7)  28.0 (9.9) | ICD-10 | Observational | Group definition, overlapping sample | Binary |
| Nordgaard 2023^13^ | Denmark | - Schizotypal disorder (n=14)  - Schizotypal disorder (n=29)  - Schizotypal disorder (n=17)  - Non-affective psychosis (n=36)  - Non-affective psychosis (n=45)  - Non-affective psychosis (n=7)  - Schizophrenia spectrum psychosis (n=35)  - OCD (n=13)  - Mixed clinical sample (n=24)  - Mixed clinical sample (n=6) | .  .  .  .  .  .  .  .  .  . | .  .  .  .  .  .  .  .  .  . | ICD-10 | Observational | Group definition, overlapping sample | Binary |
| Northoff 2021^14^ | Denmark | - Schizophrenia spectrum psychosis (n=34)  - Healthy controls (n=35) | 20 (84)  21 (60) | 22.12 (3.93)  24.06 (3.24) | DSM 5 | Observational – EEG task | Overlapping sample | Binary |
| Foerster 2024^15^ | France | - Schizophrenia spectrum psychosis (n=24) | 4 (16.7) | 31.1 (8) | Clinical/ hospital | Experimental | Overlapping sample | Binary |
| Lucarini 2022^16^ | Italy | - Schizophrenia spectrum psychosis (n=27) | . | . | DSM-5 | Observational | Overlapping sample | Binary |
| Lucarini 2024^17^ | Italy | - Schizophrenia spectrum psychosis (n=27) | 6 (22.22) | 32.15 (12.15) | DSM 5 | Observational – PPS task | Overlapping sample | Binary |
| Koren 2020^18^ | Israel | - Mixed clinical sample (n=39) | 16 (41.03) | 16.1 (1.3) | DSM-IV | Observational | Group definition | Binary |
| Baklund 2025^19^ | Norway | - CHR-P (n=27) | 16 (59.3) | 16.1 (1.2) | SIPS | Observational | Overlapping sample | Binary, Continuous |
| Haug 2012^20^ | Norway | - Schizophrenia spectrum psychosis (n=49) | 23 (46.9) | 25.8 (7.8) | DSM-IV | Observational | Overlapping sample | Binary |
| Haug 2012^21^ | Norway | - Schizophrenia spectrum psychosis (n=57) | 28 (49) | 25.4 (7.3) | DSM-IV | Observational | Overlapping sample | Binary |
| Haug 2014^22^ | Norway | - Mixed psychotic disorders (n=76) | 39 (51.32) | 25.1 (7.4) | DSM-IV | Observational | Group definition | Binary |
| Haug 2015^23^ | Norway | - Schizophrenia spectrum psychosis (n=55) | 27 (49) | 25.2 (7.3) | DSM-IV | Observational | Overlapping sample | Binary |
| Haug 2016^24^ | Norway | - Schizophrenia spectrum psychosis (n=55) | 27 (49) | 25.2 (7.3) | DSM-IV | Observational | Overlapping sample | Binary |
| Haug 2017^25^ | Norway | - Schizophrenia spectrum psychosis (n=55) | 27 (49) | 25.2 (7.3) | DSM-IV | Observational | Overlapping sample | Binary |
| Svendsen 2018^26^ | Norway | - Schizophrenia spectrum psychosis (n=35)  - Non-schizophrenia spectrum psychosis (n=21) | 18 (51.4)  10 (47.6) | .  . | DSM-IV | Observational | Scoring method, overlapping sample | Binary – item 2.12 missing |
| Svendsen 2019^27^ | Norway | - Mixed psychotic disorders (n=56) | 28 (50) | 25.2 (7.4) | DSM-IV | Observational | Group definition | Binary |
| Svendsen 2020^28^ | Norway | - Schizophrenia spectrum psychosis (n=35)  - Non-schizophrenia spectrum psychosis (n=21) | .  . | .  . | DSM-IV | Observational | Overlapping sample | Binary |
| Værnes 2021^29^ | Norway | - Mixed psychosis risk syndromes (n=32) | 11 (34.38) | 19.9 (3.8) | DSM-IV, SIPS/SOPS | Observational | Overlapping sample | Binary |
| Piani 2025^30^ | Switzerland | - Schizophrenia (n=9)  - Schizoaffective disorder (n=5)  - APPD with symptoms of SCZ (n=5)  - APPD without symptoms of SCZ (n=4)  - Substance induced psychotic disorder (n=1)  - Schizotypal disorder (n=1)  - Depression with psychotic symptoms (n=1)  - Psychosis risk syndrome (n=1) | .  .  .  .  .  .  .  . | .  .  .  .  .  .  .  . | ICD-10 | Observational - MRI | Overlapping sample | Binary |
| Bonoldi 2019^31^ | United Kingdom | - CHR-P (n=31)  - Healthy controls (n=16) | 13 (40.9)  11 (68.8) | 23.3 (4.3)  24.9 (3.3) | CAARMS | Observational | EASE scoring method | Continuous + subitems |
| Madeira 2016^32^ | United Kingdom | - Healthy controls (n=14)  - CHR-P with ABP (n=14)  - CHR-P without ABP (n=13) | 10 (71.4)  6 (42.9)  5 (23.8) | 24.21 (3.22)  24.24 (4.605)  23.25 (4.070) | CAARMS | Observational | EASE scoring method | Binary + subitems |
| Madeira 2016^33^ | United Kingdom | - Healthy controls (n=14)  - CHR-P with TS (n=13)  - CHR-P without TS (n=13) | 10 (71.4)  5 (38.5)  6 (46.2) | 24.21 (3.22)  23.00 (4.02)  24.46 (4.61) | CAARMS | Observational | EASE scoring method | Binary + subitems |

ABP = abnormal bodily phenomena; APPD = acute polymorphic psychotic disorder; CAARMS = Comprehensive Assessment of At Risk Mental States; CHR-P = clinical high-risk for psychosis; EASE = Examination of Anomalous Self-Experiences; MRI = magnetic resonance imaging; OCD = obsessive-compulsive disorder; PPS = peripersonal space; SD = standard deviation; SIPS = Structured Interview for Psychosis-Risk Syndromes; TS = Truman symptoms.

# sTable 6. Quality analysis

| **Study** | **External validity** | | | | **Internal validity** | | | | | | **Overall risk of bias score** |
| --- | --- | --- | --- | --- | --- | --- | --- | --- | --- | --- | --- |
|  | **Item 1** | **Item 2** | **Item 3** | **Item 4** | **Item 5** | **Item 6** | **Item 7** | **Item 8** | **Item 9** | **Item 10** |  |
| Baklund 2024^34^ | No | Yes | Can’t tell | Yes | Yes | Yes | Yes | Yes | No | Yes | 7 |
| Barata 2025^35^ | Can’t tell | Can’t tell | Can’t tell | Yes | Yes | Yes | Can’t tell | Yes | Can’t tell | Yes | 5 |
| Comparelli 2016^36^ | No | Yes | Yes | Yes | Yes | Can’t tell | Yes | Yes | Can’t tell | Yes | 7 |
| Davidsen 2009^37^ | Can’t tell | No | Can’t tell | Yes | Yes | Yes | Can’t tell | Yes | Can’t tell | Yes | 5 |
| Donati 2021^38^ | No | Can’t tell | No | Yes | Yes | Yes | Can’t tell | No | Can’t tell | Yes | 4 |
| Fischer-Vieler 2025^39^ | No | No | No | Yes | Yes | Yes | Yes | Yes | Can’t tell | Yes | 6 |
| Gruber 2023^40^ | Yes | Yes | No | Yes | Yes | Yes | Can’t tell | Yes | Can’t tell | Yes | 7 |
| Handest 2025^41^ | Yes | Yes | Can’t tell | No | Yes | Yes | Yes | Yes | Can’t tell | Yes | 7 |
| Haug 2012^42^ | Yes | Yes | Can’t tell | Yes | Yes | Yes | Yes | Yes | Yes | Yes | 9 |
| Haug 2023^43^ | Yes | Yes | Can’t tell | Yes | Yes | Yes | Yes | Yes | Yes | Yes | 9 |
| Koren 2016^44^ | No | Yes | No | Yes | Yes | Can´t tell | Yes | Yes | Can’t tell | Yes | 6 |
| Koren 2019^45^ | No | Yes | Yes | Yes | Yes | Yes | Yes | Yes | Can’t tell | Yes | 8 |
| Lucarini 2024^46^ | Yes | Yes | Can’t tell | Yes | Yes | Yes | Can’t tell | No | Can’t tell | Yes | 6 |
| Madeira 2017^47^ | Yes | No | Can’t tell | Yes | Yes | No | Yes | Yes | Can’t tell | Yes | 6 |
| Madeira 2019^48^ | No | Yes | Can’t tell | Yes | Yes | Yes | Yes | Yes | Yes | Yes | 8 |
| Martin 2017^49^ | No | Can’t tell | Can’t tell | Yes | Yes | Yes | Yes | No | Can’t tell | Yes | 5 |
| Monducci 2025^50^ | No | Yes | Can’t tell | Yes | Yes | Yes | Yes | Yes | Can’t tell | Yes | 7 |
| Nelson 2012^4^ | Yes | Yes | No | Yes | Yes | Yes | Yes | Yes | Can’t tell | Yes | 8 |
| Nelson 2013^51^ | Yes | Yes | No | Yes | Yes | Yes | Can’t tell | Yes | Can’t tell | Yes | 7 |
| Nelson 2018^52^ | No | Yes | Can’t tell | Yes | Yes | Can’t tell | Can’t tell | Yes | Can’t tell | Yes | 5 |
| Nelson 2020^53^ | Yes | Yes | Can’t tell | Yes | Yes | Yes | Can’t tell | Yes | Can’t tell | Yes | 7 |
| Nilsson 2020^54^ | Yes | Yes | Can’t tell | Yes | Yes | Yes | Yes | Yes | Can’t tell | Yes | 8 |
| Nordgaard & Parnas 2014^55^ | No | Yes | Yes | Yes | Yes | Yes | Yes | Yes | Can’t tell | Yes | 8 |
| Nordgaard 2015^56^ | No | Yes | Yes | Yes | Yes | Yes | Yes | Yes | Can’t tell | Yes | 8 |
| Park 2020^57^ | Yes | Yes | No | Yes | Yes | Can’t tell | Can’t tell | Yes | Can’t tell | Yes | 6 |
| Piani 2025^58^ | No | Can’t tell | Can’t tell | Yes | Yes | Yes | Can’t tell | Yes | Can’t tell | Yes | 5 |
| Raballo & Parnas 2012^59^ | No | Yes | Can’t tell | Yes | Yes | Yes | Yes | Yes | Can’t tell | Yes | 7 |
| Raballo 2016^60^ | Yes | Yes | No | Yes | Yes | Yes | Yes | Yes | Can’t tell | Yes | 8 |
| Raballo 2018^61^ | No | Yes | Yes | Yes | Yes | Can’t Yes | Yes | Yes | Can’t tell | Yes | 8 |
| Rasmussen 2020^62^ | Yes | Yes | Can’t tell | Yes | Yes | Yes | Yes | Yes | Can’t tell | Yes | 8 |
| Rasmussen 2020^63^ | No | Yes | Can’t tell | Yes | Yes | Yes | Yes | Yes | Yes | Yes | 8 |
| Rasmussen 2022^64^ | No | Yes | Can’t tell | Yes | Yes | Yes | Yes | Yes | Yes | Yes | 8 |
| Sandsten 2022^65^ | Yes | Yes | Can’t tell | Yes | Yes | Yes | Yes | Yes | Can’t tell | Yes | 8 |
| Skodlar & Parnas 2010^66^ | Can’t tell | Yes | Can’t tell | Yes | Yes | Yes | Yes | Yes | Can’t tell | Yes | 7 |
| Spark 2021^67^ | Yes | Yes | Can’t tell | Yes | Yes | Yes | Can’t tell | Yes | Can’t tell | Yes | 7 |
| Tonna 2023^68^ | No | Yes | Can’t tell | Yes | Yes | Yes | Yes | No | Can’t tell | Yes | 6 |
| Værnes 2019^69^ | Yes | Yes | No | Yes | Yes | Yes | Yes | Yes | Can’t tell | Yes | 8 |
| Zandersen & Parnas 2020^70^ | No | Yes | Can’t tell | Yes | Yes | Yes | Yes | Yes | Can’t tell | Yes | 7 |

# sTable 7. Studies included in estimates of weighted means of binary EASE scores

| Diagnostic group | Study | N | Total EASE (SD) | Domain 1 (SD) | Domain 2 (SD) | Domain 3 (SD) | Domain 4 (SD) | Domain 5 (SD) |
| --- | --- | --- | --- | --- | --- | --- | --- | --- |
| Schizophrenia spectrum psychosis | Skodlar & Parnas 2010^66^ | 25 | 18.28 (6.69) | 6.24 (2.77) | 8.68 (3.42) | 1.2 (1.47) | . | . |
|  | Haug 2012^42^ | 57 | 25.3 (9.6) | . | . | . | . | . |
|  | Raballo & Parnas 2012^59^ | 19 | 21.4 (9.6) | . | . | . | . | . |
|  | Nelson 2013^51^ | 8 | 22.5 (12.98) | 7.5 (4.63) | 8.38 (4.31) | 2 (1.93) | 1.63 (1.51) | 3.63 (2.62) |
|  | Nordgaard 2015^56^ | 31 | 21.5 (9.04) | . | . | . | . | . |
|  | Martin 2017^49^ | 28 | 15.4 (7.7) | 6.6 (3.9) | 4.8 (3) | 1.6 (1.5) | 1 (0.9) | 1.4 (1.4) |
|  | Zandersen & Parnas 2020^70^ | 6 | 19 (4.25) | . | . | . | . | . |
|  | Sandsten 2022^65^ | 35 | 25.11 (9.46) | 9.17 (2.83) | 9.09 (3.8) | 3.06 (1.83) | 1.49 (1.2) | 2.31 (1.75) |
|  | Haug 2023^43^ | 35 | .^1^ | 8.7 (3.2) | 7.5 (3.6) | 2.8 (2.1) | 1.4 (1.2) | 2.9 (2.1) |
|  | Tonna 2023^68^ | 43 | 16.5 (6.5) | 5.7 (2.8) | 6.7 (3.1) | 1.7 (1.8) | 0.4 (0.8) | 1.9 (1.8) |
|  | Lucarini 2024^46^ | 29 | 17.79 (10.15) | 6.34 (3.58) | 6.31 (3.95) | 1.9 (2.14) | 1.14 (1.51) | 2.1 (1.76) |
|  | Fischer-Vieler 2025^39^ | 17 | 12.1 (8.7) | 5.2 (4.3) | . | . | . | . |
|  | Fischer-Vieler 2025^39^ | 16 | 15.7 (7.3) | 6.6 (3.7) | . | . | . | . |
|  | Piani 2025^58^ | 15 | 10.4 (6.6) | . | . | . | . | . |
|  | Piani 2025^58^ | 5 | 9.4 (4.7) | . | . | . | . | . |
| Non-schizophrenia spectrum psychosis^2^ | Haug 2012^42^ | 21 | 6.3 (4.8) | . | . | . | . | . |
|  | Haug 2012^42^ | 13 | 11.5 (8.7) | . | . | . | . | . |
|  | Nelson 2013^51^ | 8 | 10.25 (7.15) | 4.5 (3.21) | 3.25 (2.6) | 0.34 (7.44) | 0.38 (0.52) | 1.75 (1.67) |
|  | Piani 2025^58^ | 6 | 9 (6.5) | . | . | . | . | . |
| Schizotypal disorder | Raballo & Parnas 2012^59^ | 8 | 17 (7.2) | . | . | . | . | . |
|  | Nordgaard & Parnas 2014^55^ | 22 | 17.82 (6.82) | . | . | . | . | . |
|  | Værnes 2019^69^ | 6 | 22 (3.41) | . | . | . | . | . |
|  | Nilsson 2020^54^ | 29 | 25.24 (6.42) | 9.72 (2.63) | 9.03 (2.28) | 2.24 (1.64) | 0.97 (0.87) | 3.28 (1.87) |
|  | Zandersen & Parnas 2020^70^ | 14 | 16.8 (5) | . | . | . | . | . |
|  | Rasmussen 2022^64^ | 15 | 15.4 (3.8) | . | . | . | . | . |
| CHR-P | Davidsen 2009^37^ | 11 | 5.182 (3.868) | 1.636 (2.063) | 2 (1.884) | 1.182 (1.471) | 0.182 (0.405) | 0.182 (0.603) |
|  | Koren 2016^44^ | 12 | 18.8 (7) | . | . | . | . | . |
|  | Raballo 2016^60^ | 29 | 17.31 (9.06) | . | . | . | . | . |
|  | Raballo 2018^61^ | 23 | 12 (4.5) | 6.3 (2.9) | 3.9 (2.7) | 0.9 (1.6) | 0.4 (0.6) | 0.8 (1.1) |
|  | Koren 2019^45^ | 21 | 10.76 (7.72) | . | . | . | . | . |
|  | Værnes 2019^69^ | 31 | 15.65 (8.9) | . | . | . | . | . |
|  | Park 2020^57^ | 19 | 12.5 (9.2) | . | . | . | . | . |
|  | Gruber 2023^40^ | 24 | 17.08 (6.953) | . | . | . | . | . |
|  | Baklund 2024^34^ | 27 | 16.4 (6.1) | . | . | . | . | . |
|  | Monducci 2025^50^ | 33 | 14 (6.1) | . | . | . | . | . |
| Healthy controls | Madeira 2017^47^ | 47 | 1 (1.72) | . | . | . | . | . |
|  | Madeira 2019^48^ | 24 | 1 (0.1445) | . | . | . | . | . |
|  | Park 2020^57^ | 24 | 0.4 (0.9) | . | . | . | . | . |
|  | Sandsten 2022^65^ | 35 | 0.94 (1.33) | 0.31 (0.63) | 0.34 (0.68) | 0.03 (0.17) | 0 (0) | 0.26 (0.44) |
| Single-study estimates | | | | | | | | |
| Affective disorders | Raballo & Parnas 2012^59^ | 9 | 7.36 (3.49)  7.36 (3.49)  5.7 (7.1) | . | . | . | . | . |
| Asperger’s syndrome | Nilsson 2020^54^ | 22 | 7.36 (3.49) | 4.36 (1.84) | 2.05 (1.5) | 0.27 (0.46) | 0.23 (0.43) | 0.45 (0.74) |
| Borderline personality disorder | Gruber 2023^40^ | 27 | 14.71 (7.357) | . | . | . | . | . |
| Obsessive-compulsive disorder | Rasmussen 2020^63^ | 12 | 5.33 (3.47) | . | . | . | . | . |
| Panic disorder | Madeira 2017^47^ | 47 | 13.11 (8.8) | . | . | . | . | . |

CHR-P = clinical high-risk for psychosis; SD = standard deviation.

^1^ Total score reported in study, but not used in estimate due to overlapping sample.

^2^ Includes delusional disorder or psychosis not otherwise specified (n=18), bipolar disorder with psychotic features (n=22), acute polymorphic psychotic disorder with symptoms of schizophrenia (n=6), affective disorder with psychotic features (n=1), and substance-induced psychotic disorder (n=1).

# sTable 8. Studies included in estimates of weighted means of continuous EASE scores

| Diagnostic group | Study | N | Total EASE (SD) | Domain 1 (SD) | Domain 2 (SD) | Domain 3 (SD) | Domain 4 (SD) | Domain 5 (SD) |
| --- | --- | --- | --- | --- | --- | --- | --- | --- |
| Schizophrenia spectrum psychosis | Rasmussen 2020^62^ | 26 | 71.65 (34.14) | . | . | . | . | . |
|  | Donati 2021^38^ | 10 | 78.3 (32.3) | 26.08 (13.3) | 29.9 (13) | 5.9 (6.4) | 5.1 (5) | 10.6 (5.2) |
|  | Nelson 2013^51^ | 8 | 73.5 (46.22) | 24.13 (16.43) | 27.13 (15.75) | 8.13 (7.14) | 3.88 (2.47) | 10.25 (7.63) |
| Non-schizophrenia spectrum psychosis | Spark 2021^67^ | 21 | 60.9 (27.13) | 27.05 (11.27) | 18.1 (11.16) | 6.48 (5.36) | 3 (4.13) | 6.29 (4.69) |
|  | Nelson 2013^51^ | 8 | 33.63 (17.48) | 14.13 (6.51) | 11 (7.31) | 1.38 (2.77) | 1.13 (1.13) | 6 (5.26) |
| CHR-P | Comparelli 2016^36^ | 45 | 51.09 (29.51) | 19.02 (13.42) | 24.73 (12.75) | 2.64 (3.35) | 1.71 (2.68) | 3.04 (4.03) |
|  | Værnes 2019^69^ | 31 | 52 (29) | . | . | . | . | . |
|  | Nelson 2020^53^ | 50 | 63.3 (34.65) | . | . | . | . | . |
|  | Baklund 2024^34^ | 27 | 46.9 (15.7) | . | . | . | . | . |
|  | Nelson 2012^4^ | 49 | 45.02 (26.2) | 17.76 (10.24) | 16.96 (10.83) | 2.65 (3.05) | 2.98 (2.3) | 4.67 (5.3) |
|  | Barata 2025^35^ | 18 | .^1^ | 23.7 (9.2) | 12.6 (7.3) | 2.2 (2) | 0.4 (1.2) | 2.3 (4.5) |
|  | Barata 2025^35^ | 25 | .^1^ | 31.3 (14) | 23.9 (13.7) | 7.7 (7.5) | 2.6 (3.4) | 4.5 (6.5) |
| Healthy controls | Nelson 2012^4^ | 52^2^ | 2.37 (2.45) | . | . | . | . | . |
|  | Nelson 2018^52^ | 11 | 6.45 (6.31) | . | . | . | . | . |
|  | Spark 2021^67^ | 34 | 5.32 (4.95) | 3.85 (3.67) | 1.12 (1.47) | 0.21 (0.72) | 0.03 (0.17) | 0.12 (0.41) |

CHR-P = clinical high-risk for psychosis; SD = standard deviation.

^1^ Total score reported in study, but not used in estimate due to overlapping sample.

^2^ Includes 2 controls with a diagnosis of affective disorders and 2 with other (non-affective and non-anxiety) Axis-I disorder.

# sTable 9. EASE total scores (binary) by major diagnostic categories - all eligible samples

| Major diagnostic categories | k | n | Weighted mean | Low 95% CI | High 95% CI | tau2 | I^2^ |
| --- | --- | --- | --- | --- | --- | --- | --- |
| Schizophrenia spectrum disorders^1^ | 21 | 490 | 18.40 | 16.44 | 20.35 | 17.814 | 88.9 |
| CHR-P | 10 | 230 | 13.89 | 11.35 | 16.42 | 14.499 | 88.6 |
| Non-schizophrenia spectrum psychosis^2^ | 4 | 48 | 8.64 | 5.90 | 11.38 | 3.620 | 46.7 |
| Other mental disorders^3^ | 8 | 231 | 8.27 | 5.83 | 10.71 | 10.910 | 92.5 |
| Healthy controls | 4 | 130 | 0.84 | 0.55 | 1.13 | 0.058 | 70.8 |

CHR-P = clinical high-risk state for psychosis; CI = confidence interval.

^1^ Includes schizophrenia, schizophreniform or schizoaffective disorder (n=334), schizotypal disorder (n=94), and a mixed sample containing any schizophrenia spectrum disorder (n=62).

^2^ Includes delusional disorder or psychosis not otherwise specified (n=18), bipolar disorder with psychotic features (n=22), acute polymorphic psychotic disorder with symptoms of schizophrenia (n=6), affective disorder with psychotic features (n=1), and substance-induced psychotic disorder (n=1).

^3^ Includes anxiety disorders (n=68), major depression or cyclothymic disorder (n=46), borderline personality disorder (n=27), Asperger’s syndrome (n=23), obsessive-compulsive disorder (OCD, n=12), eating disorders (n = 7), conduct disorder (n=4), mixed personality disorders (n=3), Tourette disorder (n=1), and other non-CHR and non-psychotic disorders (n=8). One study reports a mixed sample of bipolar disorder, major depression, anxiety disorders, OCD, and other personality disorders (n=32)^55^.

# sTable 10. Pairwise comparisons of EASE total scores (binary) by major diagnostic categories – all eligible samples

| Comparisons | Q | p value |
| --- | --- | --- |
| Healthy controls vs schizophrenia spectrum disorders | 68.25 | < 0.001 |
| Healthy controls vs non-schizophrenia spectrum psychosis | 60.85 | < 0.001 |
| CHR-P vs healthy controls | 46.08 | < 0.001 |
| Other mental disorders vs schizophrenia spectrum disorders | 33.33 | < 0.001 |
| Healthy controls vs other mental disorders | 20.80 | < 0.001 |
| Non-schizophrenia spectrum psychosis vs schizophrenia spectrum disorders | 14.28 | < 0.001 |
| CHR-P vs other mental disorders | 9.45 | 0.002 |
| CHR-P vs schizophrenia spectrum disorders | 7.14 | 0.007 |
| CHR-P vs non-schizophrenia spectrum psychosis | 4.20 | 0.041 |
| Non-schizophrenia spectrum psychosis vs other mental disorders | 0.10 | 0.754 |

# sTable 11. EASE total scores (binary) by major diagnostic categories – high-quality samples

| Major diagnostic categories | k | n | Weighted mean | Low 95% CI | High 95% CI | tau2 | I^2^ |
| --- | --- | --- | --- | --- | --- | --- | --- |
| Schizophrenia spectrum disorders^1^ | 13 | 329 | 20.55 | 18.64 | 22.46 | 10.014 | 83.5 |
| CHR-P | 6 | 164 | 14.29 | 12.33 | 16.25 | 4.191 | 72.0 |
| Non-schizophrenia spectrum psychosis^2^ | 2 | 34 | 8.45 | 3.43 | 13.46 | 10.060 | 74.4 |
| Other mental disorders^3^ | 6 | 157 | 6.49 | 5.68 | 7.31 | 0.253 | 24.9 |
| Healthy controls | 3 | 106 | 1.00 | 0.94 | 1.06 | 0 | 0 |

CHR-P = clinical high-risk state for psychosis; CI = confidence interval.

^1^ Includes schizophrenia, schizophreniform or schizoaffective disorder (n=173) and schizotypal disorder (n=94), and a mixed sample containing any schizophrenia spectrum disorder (n=62).

^2^ Includes delusional disorder or psychosis not otherwise specified (n=13), bipolar disorder with psychotic features (n=21).

^3^ Includes anxiety disorders (n=21), major depression or cyclothymic disorder (n=46), Asperger’s syndrome (n=23), obsessive-compulsive disorder (OCD, n=12), eating disorders (n = 7), conduct disorder (n=4), mixed personality disorders (n=3), Tourette disorder (n=1), and other non-CHR and non-psychotic disorders (n=8). One study reports a mixed sample of bipolar disorder, major depression, anxiety disorders, OCD, and other personality disorders (n=32)^55^.

# sTable 12. Pairwise comparisons of EASE total scores (binary) by major diagnostic categories – high-quality samples

| Comparisons | Q | p value |
| --- | --- | --- |
| CHR-P vs healthy controls | 651.57 | < 0.001 |
| Healthy controls vs other mental disorders | 262.94 | < 0.001 |
| Healthy controls vs schizophrenia spectrum disorders | 110.18 | < 0.001 |
| Other mental disorders vs schizophrenia spectrum disorders | 97.10 | < 0.001 |
| CHR-P vs other mental disorders | 59.19 | < 0.001 |
| Healthy controls vs non-schizophrenia spectrum psychosis | 40.61 | < 0.001 |
| Non-schizophrenia spectrum psychosis vs schizophrenia spectrum disorders | 19.58 | < 0.001 |
| CHR-P vs schizophrenia spectrum disorders | 15.37 | < 0.001 |
| CHR-P vs non-schizophrenia spectrum psychosis | 7.57 | 0.006 |
| Non-schizophrenia spectrum psychosis vs other mental disorders | 0.47 | 0.491 |

# sFigure 1. Forest plot: weighted means of total binary EASE scores across schizophrenia-spectrum disorders, non-schizophrenia spectrum psychotic disorders, other mental disorders, clinical high-risk for psychosis (CHR-P), and healthy controls – all eligible studies

**
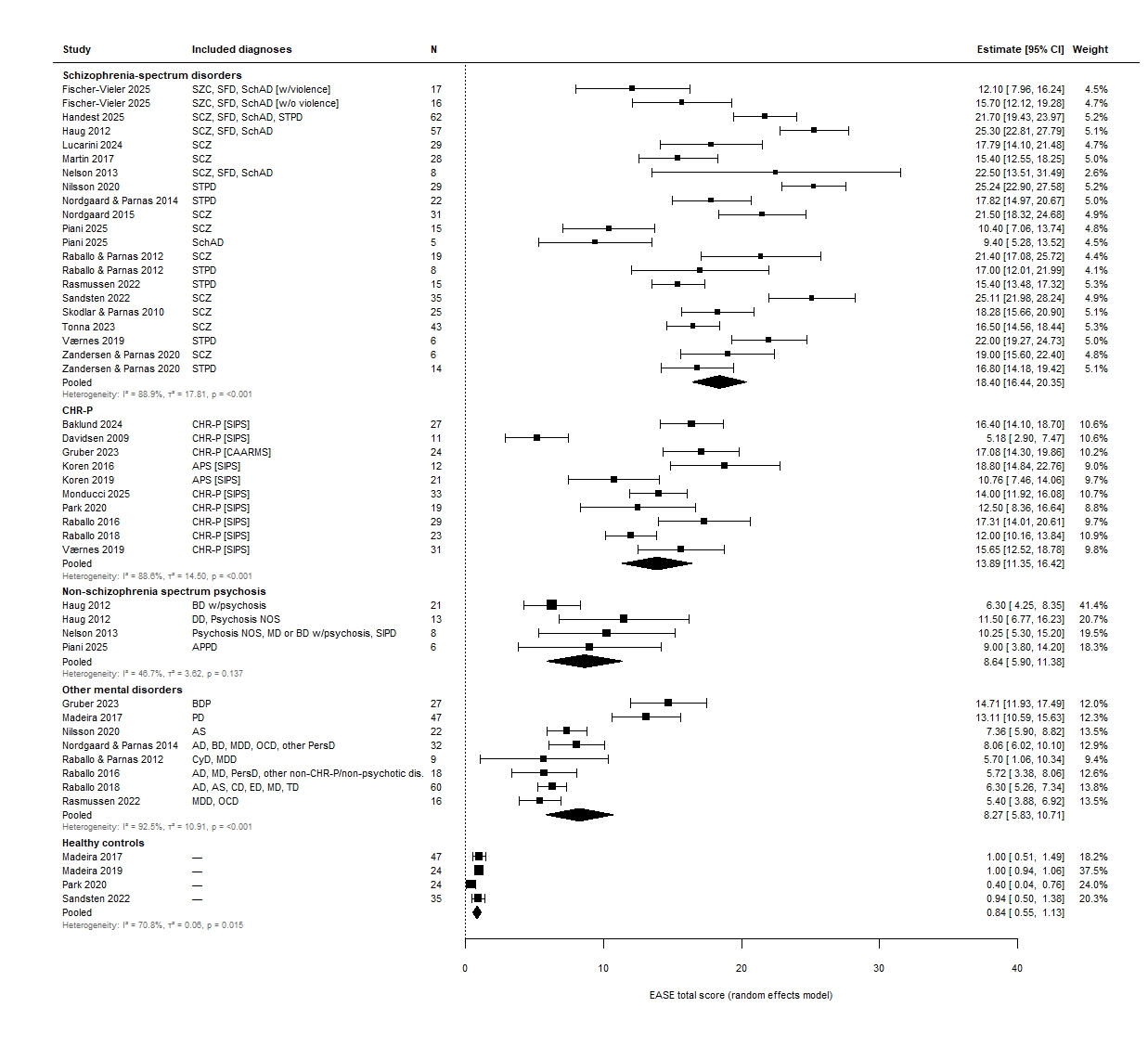
**

AD = anxiety disorder; AS = Asperger’s syndrome; APPD = acute polymorphic psychotic disorder; APS = attenuated psychosis syndrome; BD = bipolar disorder; BPD = borderline personality disorder; CAARMS = Comprehensive Assessment of At-Risk Mental States; CD = conduct disorder; CHR-P = clinical high risk for psychosis; CyD = cyclothymic disorder; ED = eating disorder; MD = mood disorder; MDD = major depressive disorder; NOS = not otherwise specified; OCD = obsessive-compulsive disorder; PersD = personality disorder; PD = panic disorder; SchAD = schizoaffective disorder; SCZ = schizophrenia; SFD = schizophreniform disorder; SIPD = substance-induced psychotic disorder; SIPS = Structured Interview for Psychosis-risk Syndromes; STPD = schizotypal (personality) disorder; TD = Tourette disorder.

# sFigure 2. Forest plot: weighted means of total binary EASE scores across schizophrenia-spectrum disorders, non-schizophrenia spectrum psychotic disorders, other mental disorders, clinical high-risk for psychosis (CHR-P), and healthy controls – high-quality samples


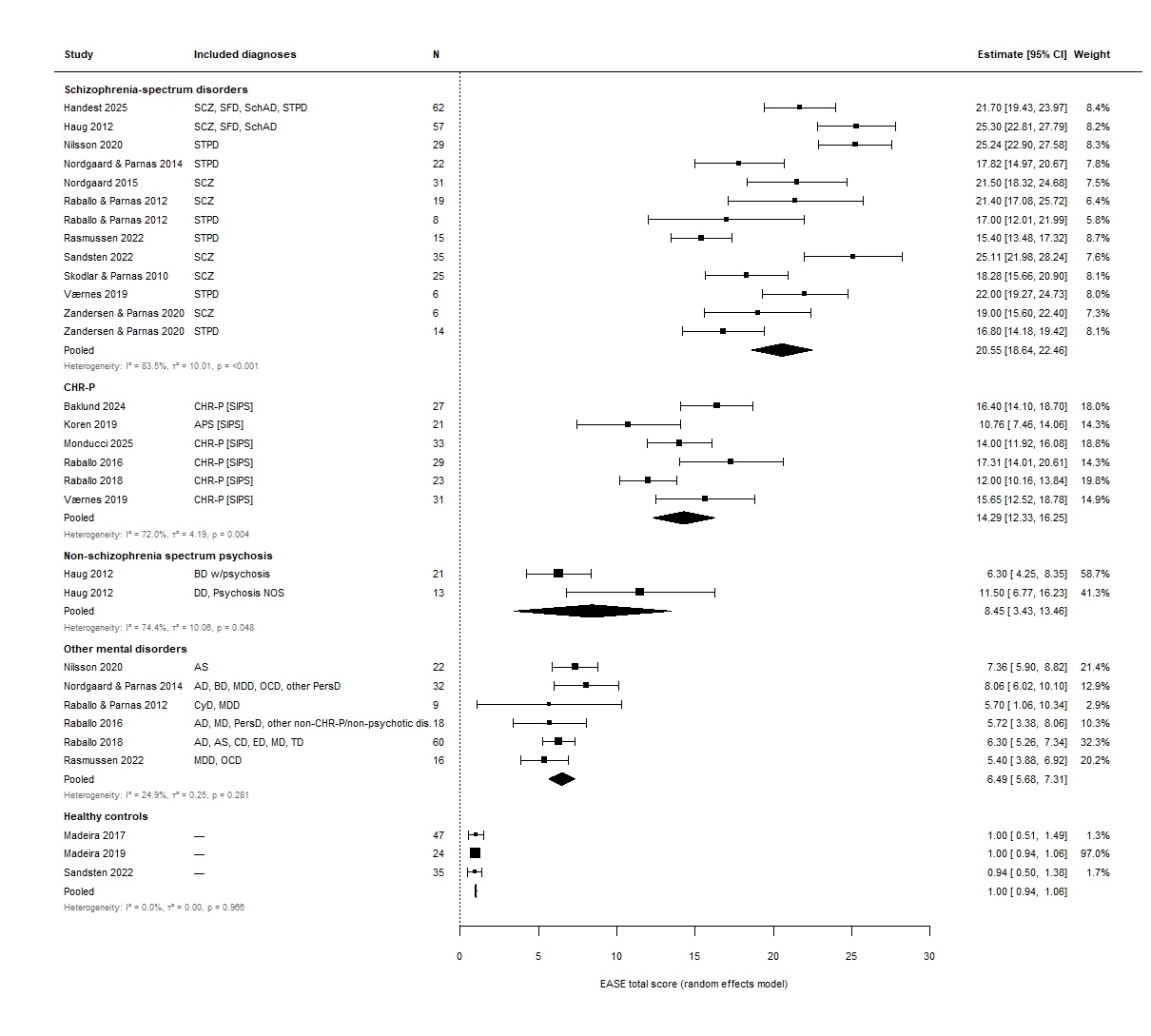


AD = anxiety disorder; AS = Asperger’s syndrome; APS = attenuated psychosis syndrome; BD = bipolar disorder; CD = conduct disorder; CHR-P = clinical high risk for psychosis; CyD = cyclothymic disorder; ED = eating disorder; MD = mood disorder; MDD = major depressive disorder; NOS = not otherwise specified; OCD = obsessive-compulsive disorder; PersD = personality disorder; SchAD = schizoaffective disorder; SCZ = schizophrenia; SFD = schizophreniform disorder; SIPS = Structured Interview for Psychosis-risk Syndromes; STPD = schizotypal (personality) disorder; TD = Tourette disorder.

# sFigure 3. Pairwise comparisons of total EASE (binary) scores across schizophrenia-spectrum disorders, non-schizophrenia spectrum psychotic disorders, other mental disorders, clinical high-risk for psychosis (CHR-P), and healthy controls – high-quality samples

**
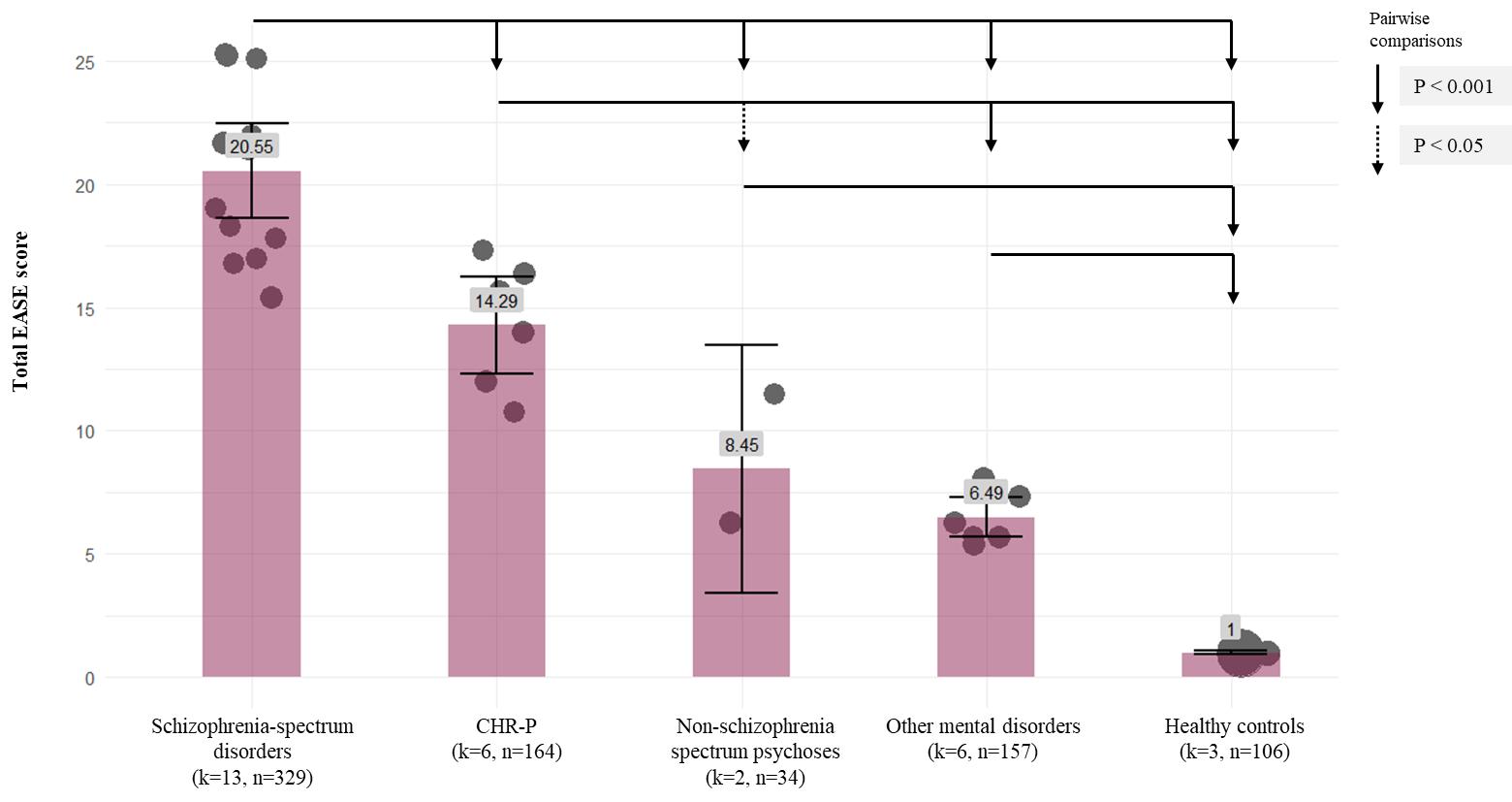
**

# sFigure 4. Forest plot of total binary EASE scores in schizophrenia-spectrum samples, stratified by study region (Denmark vs non-Denmark), used in the exploratory regional meta-regression

**
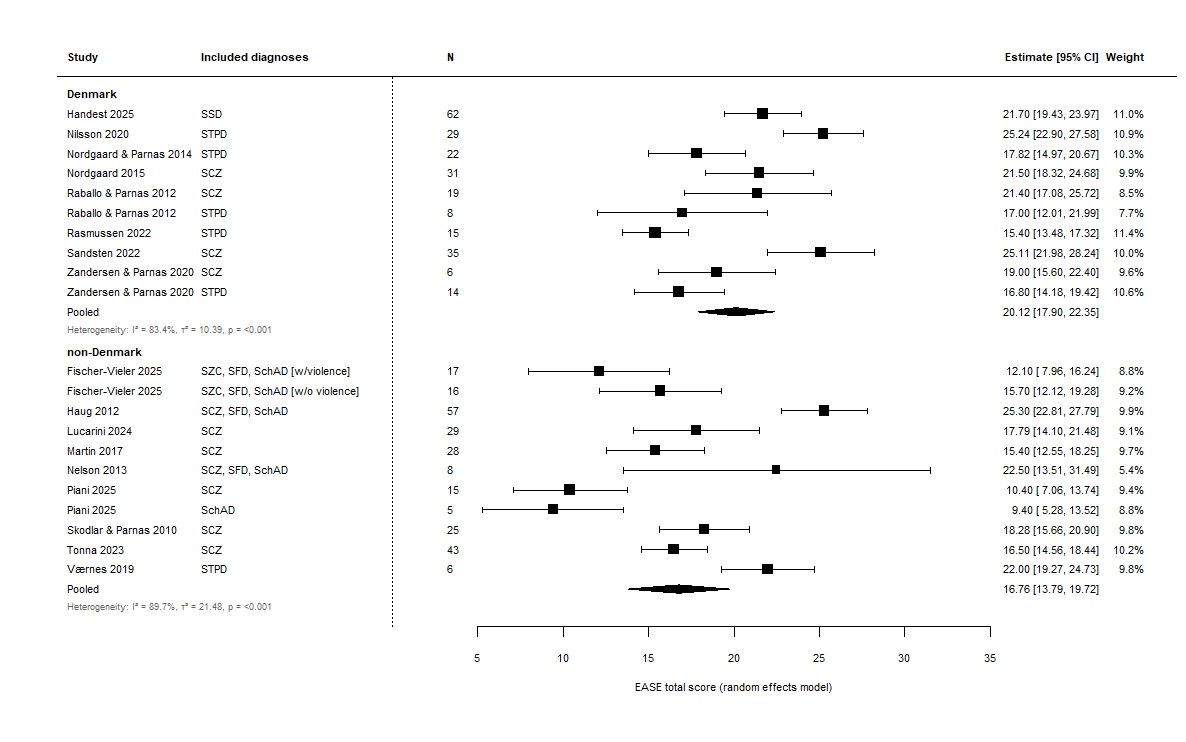
**

SchAD = schizoaffective disorder; SCZ = schizophrenia; SFD = schizophreniform disorder; STPD = schizotypal (personality) disorder.

# References

1 Fusar-Poli P, Solmi M, Brondino N, Davies C, Chae C, Politi P *et al.* Transdiagnostic psychiatry: a systematic review. *World Psychiatry* 2019; **18**: 192–207.

2 Fusar-Poli P. TRANSD recommendations: improving transdiagnostic research in psychiatry. *World Psychiatry* 2019; **18**: 361–362.

3 Møller P, Haug E, Raballo A, Parnas J, Melle I. Examination of Anomalous Self-Experience in first-episode psychosis: interrater reliability. *Psychopathology* 2011; **44**: 386–390.

4 Nelson B, Thompson A, Yung AR. Basic self-disturbance predicts psychosis onset in the ultra high risk for psychosis ‘prodromal’ population. *Schizophr Bull* 2012; **38**: 1277–1287.

5 Nordgaard J, Parnas J. A semi-structured, phenomenologically oriented psychiatric interview: descriptive congruence in assessing anomalous subjective experience and mental status. *Clin Neuropsychiatry* 2012; **9**: 123–128.

6 Arnfred SM, Raballo A, Morup M, Parnas J. Self-disorder and brain processing of proprioception in schizophrenia spectrum patients: a re-analysis. *Psychopathology* 2015; **48**: 60–64.

7 Handest R, Mølstrøm I, Henriksen MG, Nordgaard J. Duration of untreated psychosis and diagnostic delay in homeless patients with schizophrenia – a Copenhagen based clinical study. *Soc Psychiatry Psychiatr Epidemiol* 2025. doi:10.1007/s00127-025-02957-6.

8 Henriksen MG, Parnas J. Clinical manifestations of self-disorders and the gestalt of schizophrenia. *Schizophr Bull* 2012; **38**: 657–660.

9 Nordgaard J, Nilsson LS, Sæbye D, Parnas J. Self-disorders in schizophrenia-spectrum disorders: a 5-year follow-up study. *Eur Arch Psychiatry Clin Neurosci* 2018; **268**: 713–718.

10 Nordgaard J, Henriksen MG, Berge J, Nilsson LS. First-rank symptoms and self-disorders in schizophrenia. *Schizophr Res* 2019; **210**: 306–307.

11 Nordgaard J, Henriksen MG, Berge J, Siersbæk Nilsson L. Associations between Self-Disorders and First-Rank Symptoms: An Empirical Study. *Psychopathology* 2020; **53**: 103–110.

12 Nordgaard J, Gravesen-jensen M, Buch-Pedersen M, Josef P. Formal Thought Disorder and Self-Disorder: An Empirical Study. *Front Psychiatry* 2021; **12**: 1–7.

13 Nordgaard J, Berge J, Rasmussen AR, Sandsten KE, Zandersen M, Parnas J. Are self-disorders in schizophrenia expressive of a unifying disturbance of subjectivity: a factor analytic approach. *Schizophr Bull* 2023; **49**: 144–150.

14 Northoff G, Sandsten KE, Nordgaard J, Kjaer TW, Parnas J, Henriksen MG *et al.* The self and its prolonged intrinsic neural timescale in schizophrenia. *Schizophr Bull* 2021; **47**: 170–179.

15 Foerster FR, Joos E, Martin B, Coull JT, Giersch A. Self and time in individuals with schizophrenia: A motor component ? *Schizophr Res* 2024; **272**: 12–19.

16 Lucarini V, Cangemi F, Daniel BD, Lucchese J, Paraboschi F, Cattani C *et al.* Conversational metrics, psychopathological dimensions and self-disturbances in patients with schizophrenia. *Eur Arch Psychiatry Clin Neurosci* 2022; **272**: 997–1005.

17 Lucarini V, Magnani F, Ferroni F, Ardizzi M, Giustozzi F, Volpe R *et al.* Peripersonal Space Plasticity in Relation to Psychopathology and Anomalous Subjective Experiences in Individuals With Onset and Adult- ­Onset Schizophrenia. *Early Interv Psychiatry* 2025; **19**: 1–12.

18 Koren D, Tzivoni Y, Schalit L, Adres M, Reznik N, Apter A *et al.* Basic self-disorders in adolescence predict schizophrenia spectrum disorders in young adulthood: a 7-year follow-up study among non-psychotic help-seeking adolescents. *Schizophr Res* 2020; **216**: 97–103.

19 Baklund L, Røssberg JI, Melbye SA, Pesonen H, Møller P. The influence of mood and social relationships on the intensity of basic self- disturbance: an experience sampling method investigation. *Front Psychol* 2025; **16**: 1–9.

20 Haug E, Melle I, Andreassen OA, Raballo A, Bratlien U, Øie M *et al.* The association between anomalous self-experience and suicidality in first-episode schizophrenia seems mediated by depression. *Compr Psychiatry* 2012; **53**: 456–460.

21 Haug E, Øie M, Melle I, Andreassen OA, Raballo A, Bratlien U *et al.* The association between self-disorders and neurocognitive dysfunction in schizophrenia. *Schizophr Res* 2012; **135**: 79–83.

22 Haug E, Oie M, Andreassen OA, Bratlien U, Raballo A, Nelson B *et al.* Anomalous self-experiences contribute independently to social dysfunction in the early phases of schizophrenia and psychotic bipolar disorder. *Compr Psychiatry* 2014; **55**: 475–482.

23 Haug E, Øie M, Andreassen OA, Bratlien U, Nelson B, Aas M *et al.* Anomalous self-experience and childhood trauma in first-episode schizophrenia. *Compr Psychiatry* 2015; **56**: 35–41.

24 Haug E, Øie MG, Andreassen OA, Bratlien U, Romm KL, Møller P *et al.* The association between anomalous self-experiences, self-esteem and depressive symptoms in first episode schizophrenia. *Front Hum Neurosci* 2016; **10**. doi:10.3389/fnhum.2016.00557.

25 Haug E, Øie M, Andreassen OA, Bratlien U, Nelson B, Melle I *et al.* High levels of anomalous self-experience are associated with longer duration of untreated psychosis. *Early Interv Psychiatry* 2017; **11**: 133–138.

26 Svendsen IH, Øie MG, Møller P, Nelson B, Melle I, Haug E. Stability in basic self-disturbances and diagnosis in a first treated psychosis: a seven year follow-up study. *Schizophr Res* 2018; **202**: 274–280.

27 Svendsen IH, Øie MG, Møller P, Nelson B, Haug E, Melle I. Basic self-disturbances independently predict recovery in psychotic disorders: a seven year follow-up study. *Schizophr Res* 2019; **212**: 72–78.

28 Svendsen IH, Øie MG, Møller P, Nelson B, Melle I, Haug E. Basic self-disturbances are associated with Sense of Coherence in patients with psychotic disorders. *PLoS One* 2020; **15**: 1–11.

29 Værnes TG, Røssberg JI, Melle I, Nelson B, Romm KL, Møller P. Basic self-disturbance in subjects at clinical high risk for psychosis: relationship with clinical and functional outcomes at one year follow-up. *Psychiatry Res* 2021; **300**: 1–9.

30 Chiara M, Jandl M, Koenig T, Nordgaard J, Morishima Y. Pre-reflective and reflective abnormalities in cortical midline structures in schizophrenia. *Schizophr Res* 2025; **282**: 19–27.

31 Bonoldi I, Allen P, Madeira L, Tognin S, Bossong MG, Azis M *et al.* Basic self-disturbances related to reduced anterior cingulate volume in subjects at ultra-high risk for psychosis. *Front Psychiatry* 2019; **10**: 1–11.

32 Madeira L, Bonoldi I, Rocchetti M, Samson C, Azis M, Queen B *et al.* An initial investigation of abnormal bodily phenomena in subjects at ultra high risk for psychosis: their prevalence and clinical implications. *Compr Psychiatry* 2016; **66**: 39–45.

33 Madeira L, Bonoldi I, Rocchetti M, Brandizzi M, Samson C, Azis M *et al.* Prevalence and implications of Truman symptoms in subjects at ultra high risk for psychosis. *Psychiatry Res* 2016; **238**: 270–276.

34 Baklund L, Røssberg JI, Melbye SA, Møller P. Basic self-disturbance in adolescents at risk of psychosis: temporal stability investigated by the experience sampling method in a mixed method study. *BMJ Ment Heal* 2024; **27**: 1–6.

35 Barata VA, Lavoie S, Gawęda Ł, Li E, Sass LA, Koren D *et al.* The neurophenomenology of basic self-disturbance in early psychosis: Association with clinical outcome in an ultra-high risk sample. *Australas Psychiatry* 2025; **0**: 1–6.

36 Comparelli A, Corigliano V, De Carolis A, Pucci D, Angelone M, Di Pietro S *et al.* Anomalous self-experiences and their relationship with symptoms, neuro-cognition, and functioning in at-risk adolescents and young adults. *Compr Psychiatry* 2016; **65**: 44–49.

37 Davidsen KA. Anomalous self-experience in adolescents at risk of psychosis. *Psychopathology* 2009; **42**: 361–369.

38 Donati FL, Fecchio M, Maestri D, Cornali M, Derchi CC, Casetta C *et al.* Reduced readiness potential and post-movement beta synchronization reflect self-disorders in early course schizophrenia. *Sci Rep* 2021; **11**: 1–12.

39 Fischer-Vieler T, Ringen PA, Kvig E, Bell C, Hjell G, Tesli N *et al.* A phenomenological approach to violence in schizophrenia spectrum disorders: examination of anomalous self-experience. *J Forens Psychiatry Psychol* 2025; **36**: 233–246.

40 Gruber M, Alexopoulos J, Doering S, Feichtinger K, Friedrich F, Klauser M *et al.* Personality functioning and self-disorders in individuals at ultra-high risk for psychosis, with first-episode psychosis and with borderline personality disorder. *BJPsych Open* 2023; **9**: 1–9.

41 Handest R, Mølstrøm I-M, Mads Gram H, Nordgaard J. Revisiting the Relationship between Impaired Social Functioning and Psychopathology in Schizophrenia Psychiatry Research Revisiting the relationship between impaired social functioning and psychopathology in schizophrenia. *Psychiatry Res* 2025; **346**: 116389.

42 Haug E, Lien L, Raballo A, Bratlien U, Øie M, Andreassen OA *et al.* Selective aggregation of self-disorders in first-treatment DSM-IV schizophrenia spectrum disorders. *J Nerv Ment Dis* 2012; **200**: 632–636.

43 Haug E, Øie MG, Svendsen IH, Møller P, Nelson B, Melle I. A seven-year longitudinal study of the association between neurocognitive function and basic self-disorders in schizophrenia. *Front Psychol* 2023; **14**: 1–8.

44 Koren D, Lacoua L, Rothschild-Yakar L, Parnas J. Disturbances of the basic self and prodromal symptoms among young adolescents from the community: A pilot population-based study. *Schizophr Bull* 2016; **42**: 1216–1224.

45 Koren D, Scheyer R, Reznik N, Adres M, Apter A, Parnas J *et al.* Basic self-disturbance, neurocognition and metacognition: A pilot study among help-seeking adolescents with and without attenuated psychosis syndrome. *Early Interv Psychiatry* 2019; **13**: 434–442.

46 Lucarini V, Grice M, Wehrle S, Cangemi F, Giustozzi F, Amorosi S *et al.* Language in interaction: turn-taking patterns in conversations involving individuals with schizophrenia. *Psychiatry Res* 2024; **339**: 116102.

47 Madeira L, Carmenates S, Costa C, Linhares L, Stanghellini G, Figueira ML *et al.* Basic self-disturbances beyond schizophrenia: discrepancies and affinities in panic disorder - an empirical clinical study. *Psychopathology* 2017; **50**: 157–168.

48 Madeira L, Pienkos E, Filipe T, Melo M, Queiroz G, Eira J *et al.* Self and world experience in non-affective first episode of psychosis. *Schizophr Res* 2019; **211**: 69–78.

49 Martin B, Franck N, Cermolacce M, Falco A, Benair A, Etienne E *et al.* Fragile temporal prediction in patients with schizophrenia is related to minimal self disorders. *Sci Rep* 2017; **7**: 1–10.

50 Monducci E, Mammarella V, Maffucci A, Colaiori M, Cox O, Cesario S *et al.* Psychopathological Characteristics and Subjective Dimensions of Suicidality in Adolescents at Ultra High Risk (UHR) for Psychosis. *Early Interv Psychiatry* 2025; **19**: 1–11.

51 Nelson B, Thompson A, Yung AR. Not all first-episode psychosis is the same: preliminary evidence of greater basic self-disturbance in schizophrenia spectrum cases. *Early Interv Psychiatry* 2013; **7**: 200–204.

52 Nelson B, Li E, Cicero DC, Gawęda Ł, Hartmann JA, Koren D *et al.* The construct validity of the Inventory of Psychotic-Like Anomalous Self-Experiences (IPASE) as a measure of minimal self-disturbance: Preliminary data. *Early Interv Psychiatry* 2019; **13**: 686–691.

53 Nelson B, Lavoie S, Gawęda, Li E, Sass LA, Koren D *et al.* The neurophenomenology of early psychosis: an integrative empirical study. *Conscious Cogn* 2020; **77**: 1–17.

54 Nilsson M, Arnfred S, Carlsson J, Nylander L, Pedersen L, Mortensen EL *et al.* Self-disorders in Asperger syndrome compared to schizotypal disorder: a clinical study. *Schizophr Bull* 2020; **46**: 121–129.

55 Nordgaard J, Parnas J. Self-disorders and the schizophrenia spectrum: a study of 100 first hospital admissions. *Schizophr Bull* 2014; **40**: 1300–1307.

56 Nordgaard J, Revsbech R, Henriksen MG. Self-disorders, neurocognition and rationality in schizophrenia: a preliminary study. *Psychopathology* 2015; **48**: 310–316.

57 Park HY, Park K, Seo E, Koo SJ, Bang M, Park JY *et al.* Reduced activation of the ventromedial prefrontal cortex during self-referential processing in individuals at ultra-high risk for psychosis. *Aust N Z J Psychiatry* 2020; **54**: 528–538.

58 Piani MC, Jandl M, Morishima Y, Nordgaard J, Koenig T. Self-disorders in schizophrenia – and ERP study of pre-reflective and reflective self-experience. *Schizophr Res* 2025; **279**: 13–21.

59 Raballo A, Parnas J. Examination of anomalous self-experience: initial study of the structure of self-disorders in schizophrenia spectrum. *J Nerv Ment Dis* 2012; **200**: 577–583.

60 Raballo A, Pappagallo E, Dell Erba A, Lo Cascio N, Patane M, Gebhardt E *et al.* Self-disorders and clinical high risk for psychosis: An empirical study in help-seeking youth attending community mental health facilities. *Schizophr Bull* 2016; **42**: 926–932.

61 Raballo A, Monducci E, Ferrara M, Fiori Nastro P, Dario C. Developmental vulnerability to psychosis: selective aggregation of basic self-disturbance in early onset schizophrenia. *Schizophr Res* 2018; **201**: 367–372.

62 Rasmussen AR, Reich D, Lavoie S, Li E, Hartmann JA, McHugh M *et al.* The relation of basic self-disturbance to self-harm, eating disorder symptomatology and other clinical features: Exploration in an early psychosis sample. *Early Interv Psychiatry* 2020; **14**: 275–282.

63 Rasmussen AR, Nordgaard J, Parnas J. Schizophrenia-spectrum psychopathology in obsessive–compulsive disorder: an empirical study. *Eur Arch Psychiatry Clin Neurosci* 2020; **270**: 993–1002.

64 Rasmussen AR, Raballo A, Preti A, Sæbye D, Parnas J. Anomalies of imagination, self-disorders, and schizophrenia spectrum psychopathology: a network analysis. *Front Psychiatry* 2022; **12**: 1–9.

65 Sandsten KE, Wainio-Theberge S, Nordgaard J, Kjaer TW, Northoff G, Parnas J. Relating self-disorders to neurocognitive and psychopathological measures in first-episode schizophrenia. *Early Interv Psychiatry* 2022; **16**: 1202–1210.

66 Skodlar B, Parnas J. Self-disorder and subjective dimensions of suicidality in schizophrenia. *Compr Psychiatry* 2010; **51**: 363–366.

67 Spark J, Gawęda Ł, Allott K, Hartmann JA, Jack BN, Koren D *et al.* Distinguishing schizophrenia spectrum from non-spectrum disorders among young patients with first episode psychosis and at high clinical risk: The role of basic self-disturbance and neurocognition. *Schizophr Res* 2021; **228**: 19–28.

68 Tonna M, Lucarini V, Lucchese J, Presta V, Paraboschi F, Marsella F *et al.* Posture, gait and self-disorders: an empirical study in individuals with schizophrenia. *Early Interv Psychiatry* 2023; **17**: 447–461.

69 Værnes TG, Røssberg JI, Møller P. Anomalous self-experiences are strongly associated with negative symptoms in a clinical high-risk for psychosis sample. *Compr Psychiatry* 2019; **93**: 65–72.

70 Zandersen M, Parnas J. Exploring schizophrenia spectrum psychopathology in borderline personality disorder. *Eur Arch Psychiatry Clin Neurosci* 2020; **270**: 969–978.
